# Turning a green alga red: engineering astaxanthin biosynthesis by intragenic pseudogene revival in *Chlamydomonas reinhardtii*

**DOI:** 10.1111/pbi.13364

**Published:** 2020-03-31

**Authors:** Federico Perozeni, Stefano Cazzaniga, Thomas Baier, Francesca Zanoni, Gianni Zoccatelli, Kyle J. Lauersen, Lutz Wobbe, Matteo Ballottari

**Affiliations:** ^1^ Department of Biotechnology University of Verona Verona Italy; ^2^ Faculty of Biology Center for Biotechnology (CeBiTec) Bielefeld University Bielefeld Germany

**Keywords:** carotenoids, astaxanthin, microalgae, chlamydomonas

## Abstract

The green alga *Chlamydomonas reinhardtii* does not synthesize high‐value ketocarotenoids like canthaxanthin and astaxanthin; however, a β‐carotene ketolase (*Cr*BKT) can be found in its genome. *Cr*BKT is poorly expressed, contains a long C‐terminal extension not found in homologues and likely represents a pseudogene in this alga. Here, we used synthetic redesign of this gene to enable its constitutive overexpression from the nuclear genome of *C. reinhardtii*. Overexpression of the optimized *Cr*BKT extended native carotenoid biosynthesis to generate ketocarotenoids in the algal host causing noticeable changes the green algal colour to reddish‐brown. We found that up to 50% of native carotenoids could be converted into astaxanthin and more than 70% into other ketocarotenoids by robust *Cr*BKT overexpression. Modification of the carotenoid metabolism did not impair growth or biomass productivity of *C. reinhardtii*, even at high light intensities. Under different growth conditions, the best performing *Cr*BKT overexpression strain was found to reach ketocarotenoid productivities up to 4.3 mg/L/day. Astaxanthin productivity in engineered *C. reinhardtii* shown here might be competitive with that reported for *Haematococcus lacustris* (formerly *pluvialis*) which is currently the main organism cultivated for industrial astaxanthin production. In addition, the extractability and bio‐accessibility of these pigments were much higher in cell wall‐deficient *C. reinhardtii* than the resting cysts of *H. lacustris*. Engineered *C. reinhardtii* strains could thus be a promising alternative to natural astaxanthin producing algal strains and may open the possibility of other tailor‐made pigments from this host.

## Introduction

Carotenoids constitute a widely distributed group of lipid‐soluble pigments that are synthesized by plants and microorganisms (Kull and Pfander, 1995) and fulfil several important functions in photosynthetic organisms such as light harvesting, light perception and photoprotection (Mimuro and Katoh, 1991; Krieger‐Liszkay, 2005). Carotenoids are tetraterpenes, derived from eight isoprene units, (Britton, 1995) containing an extended system of conjugated double bonds that are responsible for their light harvesting and free radical scavenging capacities (Edge *et al.*, 1997). Non‐oxygenated carotenoids are named carotenes, and this subgroup contains linear (e.g. lycopene) as well as cyclic (e.g. α/β‐carotene) structures. Oxygenated derivatives of α‐ and β‐carotene are named xanthophylls. Due to their strong colour and antioxidant properties, these compounds are widely used in industry as ‘natural’ food colourants, feed additives in aquaculture and in cosmetics as well as pharmaceuticals (Hussein *et al.*, 2006; Li *et al.*, 2011; Yuan *et al.*, 2011). Animals have not been found to synthetize carotenoids naturally; however, they can structurally modify those taken up from their diet (Gerster, 1997).

Among the carotenoids, the secondary ketocarotenoid astaxanthin (3,3′‐dihydroxy‐β,β‐carotene‐4,4′‐dione) shows superior activity against reactive oxygen species (ROS) and is one of the most powerful natural antioxidants (Naguib, 2000). Astaxanthin synthesis proceeds through oxidation of both rings of β‐carotene into canthaxanthin followed by its hydroxylation (Cunningham and Gantt, 1998; Lotan and Hirschberg, 1995). Alternatively, keto groups can be added to the rings of zeaxanthin, which is derived from the hydroxylation of β‐carotene. The enzymes involved in astaxanthin synthesis are 3,3′‐β‐hydroxylase (*crtz* gene in microalgae) and 4,4′‐β‐ketolase (BKT, *crtO*gene in microalgae; Grossman *et al.*, 2004; Lotan and Hirschberg, 1995). Astaxanthin has multiple purported health benefits on biological systems due to its action against ROS (Bennedsen *et al.*, 1999; Jyonouchi *et al.*, 1995). Astaxanthin has potential uses as an antitumor agent (Kim *et al.*, 2016; Palozza *et al.*, 2009; Zhang and Wang, 2015), the prevention of cardiovascular as well as neurological diseases, and diabetes (Gross and Lockwood, 2004; Uchiyama *et al.*, 2002; Wu *et al.*, 2015). Moreover, astaxanthin can be used as human dietary supplement and in aquaculture to improve fish colour (Hussein *et al.*, 2006; Li *et al.*, 2011; Yuan *et al.*, 2011). Other ketocarotenoids like canthaxanthin, an intermediate of astaxanthin synthesis, have properties similar to astaxanthin, with high potential for use in human health applications (Miki, 1991; Møller *et al.*, 2000). With few exceptions, higher plants do not synthetize astaxanthin (Cunningham and Gantt, 2011), which is currently produced industrially from unicellular photosynthetic microalgae such as *Haematococcus lacustris* (recently renamed from *H. pluvialis*; Boussiba and Vonshak, 1991; Nakada and Ota, 2016) or, to a lesser‐extent, *Chromochloris zofingiensis* (Chen *et al.*, 2017)*. Haematococcus lacustris* is currently the main natural source of astaxanthin as it can accumulate to up to 90% of total carotenoids and 4% of cell dry weight (Bubrick, 1991) under certain environmental conditions. Astaxanthin accumulation in this alga is induced by stress conditions such as nitrogen or phosphorus starvation, high light, salt stress and elevated temperature (Boussiba and Vonshak, 1991) which stimulate the transition from motile zoospores (macrozooids) to immotile spores (aplanospores; Kobayashi *et al.*, 1997). These changes are accompanied by a degradation of the photosynthetic machinery and cessation of growth (Mascia *et al.*, 2017) as well as the formation of thick and resistant cell walls (cysts; Boussiba and Vonshak, 1991). The complexities of cellular changes to generate astaxanthin accumulation in *H. lacustris* require a two‐stage cultivation and result in a low overall productivity for the whole process. Moreover, the recalcitrance of aplanospore cell walls reduces the bio‐accessibility of astaxanthin and makes mechanical disruption necessary in order to release astaxanthin for human or animal consumption (Kang and Sim, 2008), a process which increases production process costs.

Given these limitations, genetic engineering approaches have been undertaken to enable astaxanthin production in different biotechnological host organisms in order to generate suitable alternatives to traditional *H. lacustris* production processes. Astaxanthin synthesis has indeed been demonstrated in many different organisms such as fermentative bacteria (Henke *et al.*, 2016; Park *et al.*, 2018) as well as photosynthetic cyanobacteria (Harker and Hirschberg, 1997), and eukaryotic hosts including yeasts (Kildegaard *et al.*, 2017; Miura *et al.*, 1998) and higher plants (Harada *et al.*, 2014; Hasunuma *et al.*, 2008; Huang *et al.*, 2013; Jayaraj *et al.*, 2008; Mann *et al.*, 2000; Nogueira *et al.*, 2017; Stalberg *et al.*, 2003; Zhong *et al.*, 2011) by the transgenic expression of keto‐ and hydroxylases. The results obtained were promising but with limited, industrial relevance due to the high costs of cultivation of these organisms and/or low productivity. Even if high production yields of astaxanthin have been reported upon heterotrophic cultivation of different microorganisms, the possibility to produce ketocarotenoids in photoautotrophic systems has a strong advantage in terms of sustainability, by consuming CO_2_ and avoiding the costs of reduced carbon sources used in heterotrophic cultivation. In order to develop a sustainable alternative to traditional astaxanthin production, we sought to engineer the common freshwater microalga *Chlamydomonas reinhardtii* to constitutively produce astaxanthin and canthaxanthin. In contrast to previous attempts by others (Leon *et al.*, 2007; Tan *et al.*, 2007; Zheng *et al.*, 2014), our approach is based on the synthetic redesign and revival of an endogenous yet inactive (pseudogene) β‐carotene ketolase sequence present in the nuclear genome of *C. reinhardtii*. Strains resulting from the application of this strategy generated astaxanthin, exhibited reddish‐brown phenotypes and reached productivities comparable to *H. lacustris* cultivation without many of its natural process constraints.

## Results

### Analysis of *Chlamydomonas reinhardtii* bkt gene

**Figure 1:**
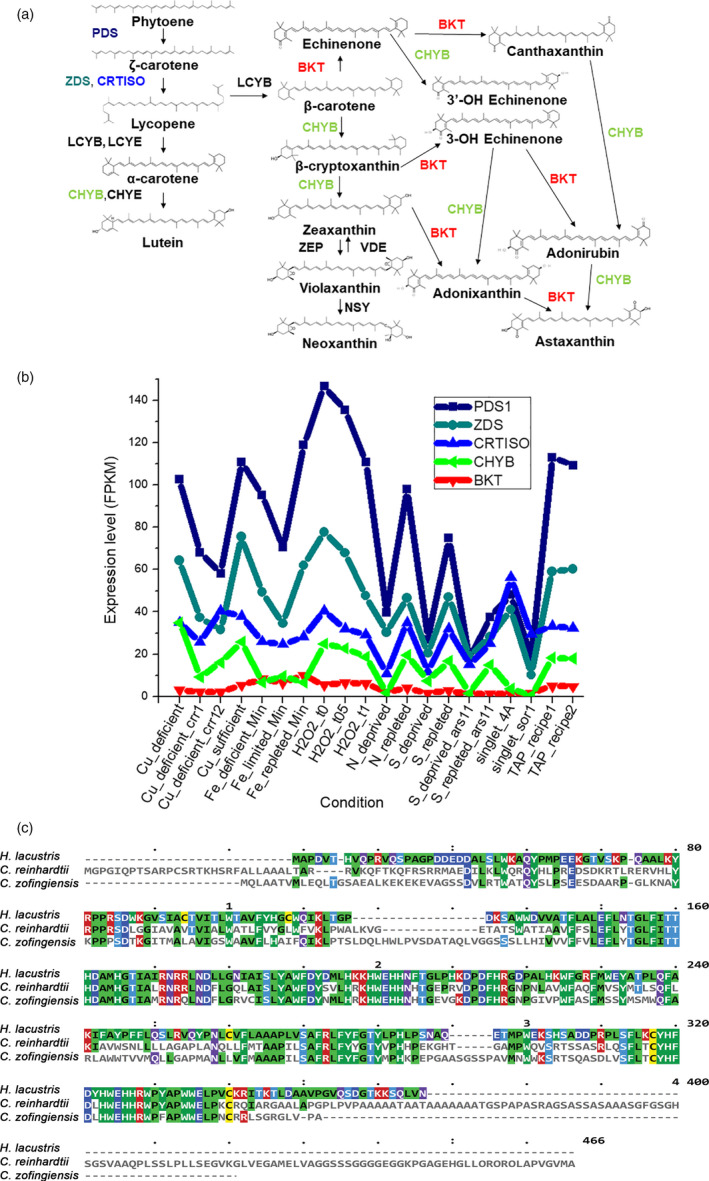
The native BKT of *C. reinhardtii* and its potential role in astaxanthin production. (a) Schematic of the carotenoid pathway towards astaxanthin biosynthesis, according to Misawa *et al. *(1995) and Alvarez *et al. *(2006). Only major carotenoids are indicated. Name of enzymes are reported. BKT, carotene β‐ketolase; CHYB, carotene β‐hydroxylase; CHYE, carotene ε‐hydroxylase; CRTISO, carotenoid isomerase; LCYB, lycopene β‐cyclase; LCYE, lycopene ε‐cyclase; NSY, neoxanthin synthase; PDS, phytoene desaturase; VDE, violaxanthin de‐epoxidase; ZDS, ζ‐carotene desaturase; ZEP, zeaxanthin epoxidase. (b) Gene expression level of *Cr*BKT native gene compared to other carotenoid biosynthetic genes. Expression levels in different conditions were retrieved from ChlamyNET database http://viridiplantae.ibvf.csic.es/ChlamyNet/. (c) Protein sequence alignment of BKT from different algae. BKT protein sequences from *Chlamydomonas reinhardtii*, *Haematococcus lacustris* and *Chlamydomonas zofingensis* were aligned by multiple alignment highlighting the long C‐terminal amino acid extension of *Cr*BKT. Colour code is on the base of consensus.

Pathways for the synthesis of carotenoids and xanthophylls in *C. reinhardtii* have already been characterized in previous studies and are depicted in Figure 1 (Lohr *et al.*, 2005). Although astaxanthin accumulation has never been reported in *C. reinhardtii* in any condition (Lohr *et al.*, 2005), a putative *Cr*BKT enzyme (Uniprot Q4VKB4) can be found in its nuclear genome (Merchant *et al.*, 2007). *Cr*BKT has indeed been previously reported to efficiently convert β‐carotene and zeaxanthin into astaxanthin when expressed in engineered *Escherichia coli* cells, even in the absence of the CrtZ hydroxylase (Huang *et al.*, 2013; Park *et al.*, 2018; Zhong *et al.*, 2011).

**Figure 2:**
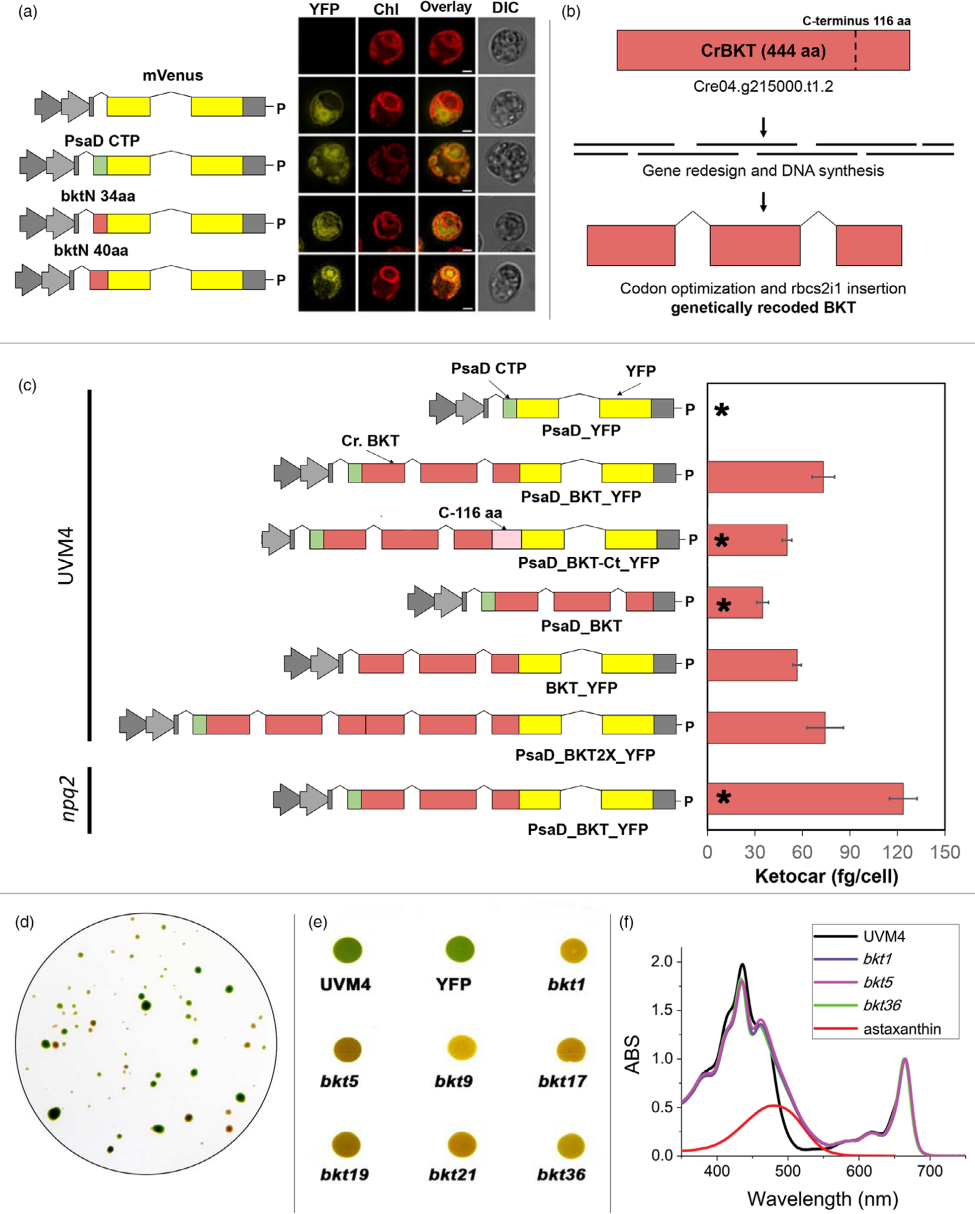
Synthetic*Cr*BKT redesign, expression vectors for *Chlamydomonas* *reinhardtii* transformation and phenotypic change in expression lines. (a) Analysis of endogenous *Cr*BKT transit peptide was performed by fusion of two different amino acid lengths of the targeting peptide to the YFP reporter. A cytoplasmic and chloroplast‐targeted control is shown, and the latter is mediated by the previously characterized PsaD chloroplast targeting peptide. YFP fluorescence (YFP), chlorophyll autofluorescence (Chl), merger of these two channels and differential interference contrast (DIC) images are shown. Scale bar represents 2 µm. (b) The optimized CrBKT sequence was built by gene synthesis after *in silico* design using codon optimization and systematic spreading of the rbcs2 intron 1 sequence to minimize exon lengths as previously described to enable robust transgene expression (Baier *et al.*, 2018b). (c) Schematic overview of all expression vectors used in this work and their respective ketocarotenoid accumulation efficiencies. All gene expression cassettes use the Hsp70A/Rbcs2 hybrid promoter containing rbcs2 intron 1 and its 5′ untranslated region as previously described (Lauersen *et al.*, 2015). PsaD_YFP: YFP localized to the chloroplast by the PsaD transit peptide. PsaD_BKT_YFP: BKT fused to YFP targeted by PsaD transit peptide. PsaD_BKT_cterm_YFP_Paro: same as previous vector with the addition of the 116 C‐terminal amino acid coding extension of the CrBKT gene. BKT_YFP_Paro: BKT fused to YFP targeted by endogenous transit peptide. PsaD_BKT: CrBKT targeted into chloroplast by PsaD transit peptide without the YFP reporter. PsaD_2xBKT_YFP: Two copies of BKT coding sequence were put in frame in order to generate a fused protein carrying two BKT and YFP. For both *BKT* copies, sequence coding for first 40 aa was removed. All proteins expressed carry a strepII affinity tag (WSHPQFEK*) on the C‐terminus. All the constructs were used to transform UVM4 strain (Neupert *et al.*, 2009b). PsaD_BKT_YFP construct was used to transform the *npq2*mutant strain, a *C. reinhardtii* strain mutated on the gene encoding for zeaxanthin epoxidase, resulting into constitutive accumulation of zeaxanthin (Niyogi *et al.*, 1997a). Selection was achieved for all constructs with the AphVIII paromomycin (P) resistance cassette of the pOpt vector backbone. Ketocarotenoid accumulation per cell is expressed as mean ± SD (*n* = 5). The significantly different value from the one obtained with PsaD BKT_YFP construct is marked with an asterisk (*) (*P* < 0.05). (d) Orange/red phenotypes of *C. reinhardtii* cells expressing CrBKT_YFP recovered after transformation and selection on solid medium. (e) Image of UVM4 and transformed cells spotted on TAP agar and grown at 100 µmol/m^2^/s; YFP represents a strain transformed with PsaD_YFP as a control, and *bkt* are lines transformed with CrBKT_YFP. (f) Spectra of acetone‐extracted pigments from UVM4 and three select *bkt* lines. Spectra are normalized to absorption in Qy region. Spectrum of astaxanthin is shown as reference in red.

The native expression level of *CrBKT* gene was thus analysed over a wide range of growth conditions using *C. reinhardtii* RNAseq databases (Romero‐Campero *et al.*, 2016). These investigations revealed a very low expression level in any of the tested conditions compared to other genes involved in carotenoid biosynthesis (Figure 1b). An *in silico* analysis of the *Cr*BKT amino acid sequence revealed an overall high degree of conservation, when compared to other BKT enzymes. A peculiarity of the sequence, however, is the presence of a 116 amino acid C‐terminal extension, which is not present in other BKT sequences from other organisms (Lohr *et al.*, 2005; Figure 1c). A putative chloroplast transit peptide was predicted on the N‐terminus of the *Cr*BKT, which was tested for its ability to enable chloroplast import of recombinant mVenus yellow fluorescent protein (hereafter, YFP) in *C. reinhardtii*. While the first 40 N‐terminal residues of *Cr*BKT were sufficient for the import of YFP into the chloroplast, a smaller sequence only comprising the first 34 residues from the N‐terminus was not (Figure 2a).

### Intragenic expression of *Chlamydomonas reinhardtii* β‐ketolase

The low native expression rates of *Cr*BKT led us to consider this may be a pseudogene with a residual expression level too low to have an impact on cellular physiology or expression only under some condition not known specific to its habitat or lifecycle. Given that the gene had been successfully expressed in *E. coli* and was shown to functionally convert carotenoids into astaxanthin (Zhong *et al.*, 2011), we decided to investigate whether the gene could be revived by synthetic redesign. Recently, we demonstrated that transgenes could be optimized for expression from the algal nuclear genome by codon optimization and systematic incorporation of the first intron of the *C. reinhardtii* RuBisCO small subunit II gene to mimic host regulatory structures (Baier *et al.*, 2018b). We have recently used this strategy for overexpression of numerous foreign genes (Lauersen *et al.*, 2016; Lauersen *et al.*, 2018; Wichmann *et al.*, 2018), as well as re‐coding and overexpression of the endogenous photodecarboxylase (*Cr*FAP; Yunus *et al.*, 2018) for various metabolic engineering activities in this host. Here, the amino acid sequence of the *Cr*BKT was used to generate an optimized synthetic algal transgene, employing the same optimization strategy (Figure 2b). The optimized gene was first generated by omitting the 116 aa C‐terminal extension as this is absent from all other gene homologues in other organisms (Figure 1c) and may influence its activity. The optimized synthetic *Cr*BKT was then cloned into the pOpt2_PsaD_mVenus_Paro vector for expression (Figure 2c; Wichmann *et al.*, 2018). In order to facilitate chloroplast import, the N‐terminus of the BKT coding sequence was fused to the chloroplast transit peptide from photosystem I subunit D (PsaD), which has been already reported to be functional for chloroplast import in different conditions (Lauersen *et al.*, 2015; Rasala *et al.*, 2013). The coding sequence of YFP was left in the vector to generate a fusion at the 3′ end of *Cr*BKT. The construct obtained (PsaD_BKT_YFP, Figure 2c) was then used to transform *C. reinhardtii* UVM4, a strain which has been mutated to enable more reliable transgene expression from the nuclear genome (Neupert *et al.*, 2009).

*Chlamydomonas reinhardtii* colonies recovered on plates after transformation using the PsaD_BKT_YFP construct exhibited clear changes in colour, from its native green to reddish‐brown (Figure 2d). The accumulation of recombinant *Cr*BKT protein in transformed cells was verified by immunoblot developed using an antibody recognizing the fused YFP (Figure S1). *Chlamydomonas reinhardtii* cells expressing *Cr*BKT appeared similar in shape and size under microscopy analysis compared to the parental strain UVM4, but with a reddish colour (Figure S2). PsaD_BKT_YFP expression lines were screened for the highest accumulation of ketocarotenoids, first by selection of colonies based on intensity of red colour and then by acetone extraction and spectral analysis (Figure 2e,f). Spectra of pigments from *bkt* lines, extracted with acetone, were found to exhibit a shoulder above 500 nm which was not present in the parental control. This shoulder corresponds to the absorption peak of ketocarotenoids, as astaxanthin (Figure 2f). Three lines exhibiting the darkest red phenotype and the largest shoulder in spectral analysis were *bkt1*, *bkt5* and *bkt36*. The content of total ketocarotenoids accumulated in the cells was estimated to be up to 73.2 ± 3.7 fg/cell by spectral analysis (see Section Methods for further details). In order to possibly improve the expression and activity, further constructs were generated with modifications in the orientation and extensions of the BKT (Figure 2c; UVM4). Many variations of this original expression construct were implemented; however, none resulted in improved astaxanthin production over the PsaD_BKT_YFP vectors (Figure 2c). First, the 116 aa extension removed at the C‐terminus of *Cr*BKT was reinserted in the vector PsaD_BKT‐Ct_YFP and this resulted in an average level of ketocarotenoids ~35% lower compared to the complete version of *Cr*BKT. The YFP coding sequence was also removed from the C‐terminus of *Cr*BKT (PsaD_BKT), in order to evaluate a possible negative effect due to the presence of YFP at the C‐terminus of the protein. The mutants obtained showed a pale red coloration, and ketocarotenoids were present although to a much lower level than in the mutants obtained with *Cr*BKT fused with YFP. A vector coding for a truncated *Cr*BKT C‐terminus fused with YFP and only using the native *Cr*BKT chloroplast targeting sequence without the PsaD‐CTP was also prepared (BKT‐YFP) resulting in ~20% lower ketocarotenoid production to the original construct. Finally, two *Cr*BKT gene copies were fused together in order to enhance the number of catalytic sites (PsaD_BKT2x_YFP) as this strategy had been previously shown beneficial for a sesquiterpene synthase in this host (Lauersen *et al.*, 2016). However, the strains obtained from this construct showed similar amounts of ketocarotenoids as the single *Cr*BKT construct (Figure 2c).

The *C. reinhardtii npq2* mutant contains a knockout mutation in the zeaxanthin epoxidase (ZEP, Figure 1a) and is unable to synthetize violaxanthin and neoxanthin; therefore, it accumulates zeaxanthin as a terminal carotenoid species. Zeaxanthin is one of the carotenoid substrates for the *Cr*BKT enzyme (Zhong *et al.*, 2011). Therefore, to determine whether ketocarotenoid yields might be higher in this host, the PsaD_BKT_YFP vector was used to transform the *npq2* mutant (Figure 2c; npq2). After transformation and selection, three lines (*n2bkt1*, *n2bkt11* and *n2bkt12*) were selected for strong reddish phenotype and analysed with the same procedure used for *bkt* lines. The ketocarotenoid content per cell of these strains was indeed increased compared to the *bkt* lines obtained in UVM4 background (Figure 2c). Therefore, strains *bkt1/5/36* and *n2bkt1/11/12*, obtained by transformation with construct PsaD_BKT_YFP in the UVM4 and *npq2* backgrounds, respectively, were used for further investigations.

### The presence of ketocarotenoids does not perturb algal growth

**Figure 3:**
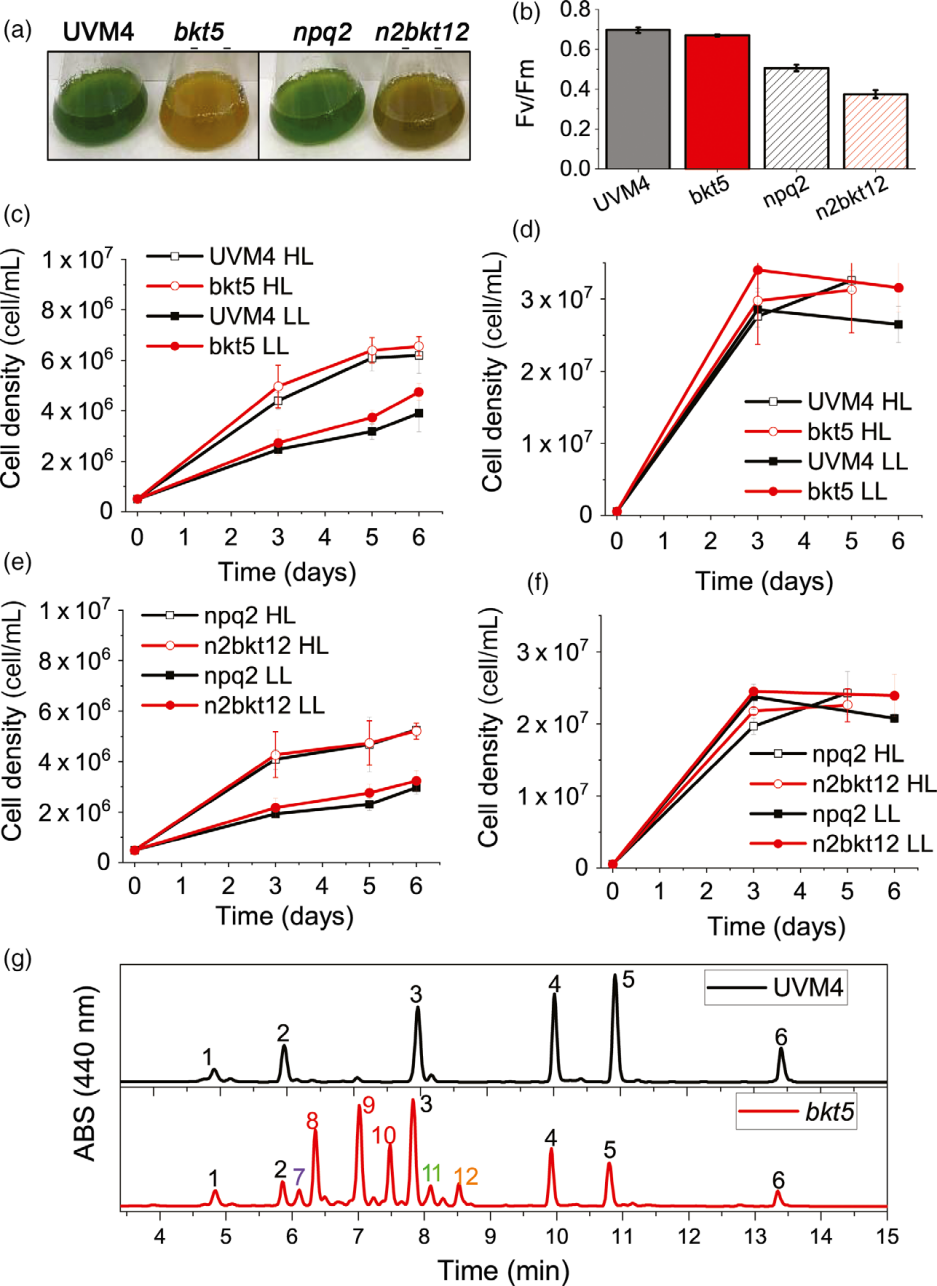
Impact of astaxanthin accumulation in *Chlamydomonas reinhardtii* UVM4 and *npq2* backgrounds on growth (a) Cultures of UVM4, *bkt5*, *npq2* and *n2bkt12* grown in autotrophy at 100 µmol/m^2^/s exhibit striking differences in colour. (b) Fv/Fm of UVM4, *bkt5*, *npq2* and *n2bkt12* grown in autotrophy at 100 µmol/m^2^/s. (c,d) Growth curves of UVM4 and *bkt5* strain in autotrophy (c, HS medium) or mixotrophy (d, TAP medium) in low light (LL, 100 µmol/m^2^/s) or high light (HL, 500 µmol/m^2^/s) conditions. (e,f) Growth curves of *npq2* and *n2bkt5* strain in autotrophy (e, HS medium) or mixotrophy (f, TAP medium) in low light (LL, 100 µmol/m^2^/s) or high light (HL, 500 µmol/m^2^/s) conditions. (g) Representative HPLC of pigments from UVM4 (black) and mutant *bkt5* (red). 1: neoxanthin, 2: violaxanthin; 3: lutein; 4: chlorophyll b; 5: chlorophyll a; 6 β‐carotene; 7: unidentified; 8: 3S,3′S trans‐astaxanthin, 9: 3S,3′S 9‐cis‐astaxanthin, 10: 3S,3′S 13‐cis‐astaxanthin11: adonirubin, 12: canthaxanthin;. Data are expressed as mean ± standard deviation (*n* = 3). In panels b–f, statistically different values comparing UVM4 vs. *bkt5*and *npq2*vs. *n2bkt12*are indicated with *(*P* < 0.05).

The influence of the presence of astaxanthin and ketocarotenoids in *C. reinhardtii* was evaluated by cultivating selected transformant lines and their parental strains (UVM4, *npq2*) in 20‐mL flasks for one week in photoautotrophy (CO_2_) or mixotrophy (acetate; Harris and Harris, 2008) at 100 or 500 µmol/m^2^/s. Although *bkt* and *n2bkt* lines exhibited reddish‐brown phenotypes, they were nevertheless able to grow even in photoautotrophic conditions (Figure 3a). Both *bkt* and *n2bkt* lines are indeed photosynthetically active: in the case of *bkt5*, a similar quantum yield of photosystem II (Fv/Fm) was measured compared to its parental strain (UVM4), while in the case of only *n2bkt*, a slight decrease of Fv/Fm was evident compared to its background *npq2.* Reduced Fv/Fm in *npq2* background compared to UVM4 is consistent with previous findings owing to the overaccumulation of zeaxanthin in this strain (Couso *et al.*, 2012): further decrease of Fv/Fm in *n2bkt12* might be related to a partial destabilization of photosynthetic apparatus due to substitution of xanthophylls with ketocarotenoids, as previously reported in the case of *H. lacustris* (Mascia *et al.*, 2017). In order to investigate a possible effect in biomass productivity related to ketocarotenoid accumulation, this was evaluated in autotrophic and mixotrophic conditions (Figure 3c–f). At either light intensity tested, growth in mixotrophy was faster compared to the autotrophic cultivation for any strain tested (Figure 3c–f). Cell dry weight was measured at stationary phase for all the genotypes (Table 1). In both TAP and HS medium, growth of *npq2* strains was slower compared to UVM4, even if the final biomass harvested was similar although in some cases statistically different (Table 1). Transformant lines exhibited biomass accumulation similar to their respective parental lines, with the only exception of an increased biomass yield in the case of *bkt5*grown in control light in mixotrophy, when compared to its background UVM4. Similar photoautotrophic biomass accumulation in strains containing astaxanthin and parentals indicated that the presence of astaxanthin does not impair algal growth.

**Table 1:** Pigment content of UVM4, *npq2*, *bkt5* and *n2bkt12* lines

Growth	Genotype	chl pg/cell	chl/car	asta/car	keto/car	asta fg/cell	keto fg/cell	dry weight (g/L)	Car/100 chl						
									neo	viola	lute	zea	b car	asta	keto
LL HS	UVM4	0.95 ± 0.09^a^	2.70 ± 0.21^a^	—	—	—	—	0.14 ± 0.01^a^	3.8 ± 0.5^a^	7.5 ± 1.3^a^	21.4 ± 3.9^a^	—	4.3 ± 0.2^a^	—	—
	*bkt5*	0.19 ± 0.04^b^	1.81 ± 0.14^b,c^	0.49 ± 0.02^a^	0.75 ± 0.02^a^	34.7 ± 3.3^a^	52.1 ± 5.8^a^	0.19 ± 0.02^b^	1.5 ± 0.2^b^	1.2 ± 0.2^b^	9.0 ± 1.5^b^	—	2.1 ± 0.1^b^	27.2 ± 3.1^a^	41.5 ± 4.1^a^
	*npq2*	0.67 ± 0.20^c^	2.49 ± 0.67^b^	—	—	—	—	0.15 ± 0.02^a^	—	—	18.6 ± 4.5^a^	16.0 ± 4.3^a^	5.6 ± 0.5^c^	—	—
	*n2bkt12*	0.25 ± 0.07^b^	1.42 ± 0.38^c^	0.62 ± 0.11^a^	0.80 ± 0.09^a^	72.5 ± 14.5^b^	92.7 ± 12.2^b^	0.14 ± 0.03^a^	—	—	11.3 ± 2.5^b^	3.5 ± 0.9^b^	1.8 ± 0.1^d^	46.6 ± 17.7^a^	59.6 ± 19.3^a^
LL TAP	UVM4	1.41 ± 0.34^a^	2.81 ± 0.11^a^	—	—	—	—	0.56 ± 0.05^a^	4.9 ± 2.1^a^	8.7 ± 2.9^a^	16.8 ± 1.1^a^	—	5.3 ± 2.1^a^	—	—
	*bkt5*	0.38 ± 0.08^b^	2.39 ± 0.09^b^	0.37 ± 0.03^a^	0.69 ± 0.03^a^	40.6 ± 3.6^a^	72.9 ± 3.5^a^	0.65 ± 0.01^b^	1.5 ± 0.6^b^	2.1 ± 0.6^b^	8.6 ± 0.5^b^	—	1.0 ± 0.3^b^	15.6 ± 1.3^a^	28.8 ± 1.3^a^
	*npq2*	1.26 ± 0.49^a^	2.80 ± 0.03^a^	—	—	—	—	0.69 ± 0.04^b^	—	—	12.3 ± 1.3^c^	19.3 ± 2.0^a^	4.0 ± 1.2^a^	—	—
	*n2bkt12*	0.72 ± 0.09^c^	2.45 ± 0.03^b^	0.42 ± 0.03^a^	0.65 ± 0.03^a^	87.3 ± 2.0^b^	130.4 ± 0.7^b^	0.65 ± 0.01^b^	—	—	13.1 ± 1.2^c^	1.6 ± 0.2^b^	0.6 ± 0.2^b^	17.3 ± 0.9^a^	26.6 ± 0.9^a^
HL HS	UVM4	0.87 ± 0.21^a^	2.54 ± 0.24^a^	—	—	—		0.14 ± 0.01^a^	3.7 ± 0.9^a^	7.5 ± 1.8^a^	21.2 ± 8.3^a^	2.8 ± 0.4^a^	4.2 ± 1.1^a^	—	—
	*bkt5*	0.09 ± 0.02^b^	0.93 ± 0.09^b^	0.47 ± 0.10^a^	0.75 ± 0.07^a^	30.9 ± 10.7^a^	47.5 ± 11.4^a^	0.13 ± 0.06^a^	2.5 ± 0.5^a^	2.1 ± 0.5^b^	16.0 ± 5.7^a^	6.0 ± 0.7^b^	2.3 ± 1.0^b^	51.5 ± 14.4^a^	81.5 ± 13.1^a^
	*npq2*	0.60 ± 0.24^a^	2.12 ± 0.73^a^	—	—	—	—	0.18 ± 0.04^a^	—	—	20.2 ± 8.0^a^	22.0 ± 4.2^c^	4.9 ± 1.6^a,c^	—	—
	*n2bkt12*	0.08 ± 0.03^b^	0.66 ± 0.22^b^	0.39 ± 0.07^a^	0.76 ± 0.04^a^	53.1 ± 9.4^b^	99.0 ± 8.2^b^	0.14 ± 0.03^a^	—	—	17.2 ± 7.4^a^	4.7 ± 2.1^b^	2.8 ± 0.4^b,c^	42.2 ± 8.1^a^	80.2 ± 11.5^a^
HL TAP	UVM4	1.04 ± 0.17^a^	2.51 ± 0.34^a^	—	—	—	—	0.71 ± 0.05^a^	4.8 ± 1.1^a^	10.4 ± 2.3^a^	17.7 ± 3.2^a^	1.6 ± 0.6^a,b^	5.4 ± 1.6^a^	—	—
	*bkt5*	0.21 ± 0.03^b^	1.80 ± 0.24^b^	0.42 ± 0.01^a^	0.74 ± 0.01^a^	33.6 ± 4.7^a^	57.3 ± 7.5^a^	0.68 ± 0.03^a,b^	1.7 ± 0.4^a^	1.8 ± 0.3^b^	9.6 ± 1.6^b^	1.3 ± 0.4^a^	1.2 ± 0.3^b^	23.8 ± 2.8^a^	41.5 ± 5.3^a^
	*npq2*	0.98 ± 0.16^a^	2.39 ± 0.29^a^	—	—	—	—	0.64 ± 0.07^b,c^	—	—	14.7 ± 2.4^c^	22.2 ± 5.4^c^	5.0 ± 0.2^a^	—	—
	*n2bkt12*	0.47 ± 0.07^c^	1.67 ± 0.20^b^	0.49 ± 0.08^a^	0.73 ± 0.07^a^	94.9 ± 16.3^b^	137.5 ± 17.2^b^	0.62 ± 0.02^c^	—	—	14.7 ± 2.2^c^	2.2 ± 0.5^b^	0.9 ± 0.1^b^	30.0 ± 8.1^a^	44.1 ± 9.4^a^

Pigment content determined in cells grown at 100 (LL) or 500 (HL) µmol/m/s in HS and TAP medium for 1 week starting from 5 × 10^5^ cells/mL. Acronyms indicate chlorophyll (chl), total carotenoid (car), astaxanthin (asta), total ketocarotenoid (keto), neoxanthin (neo), violaxanthin (viola), zeaxanthin (zea) and β‐carotene (β car). Chlorophyll per cell is expressed as picograms (pg), and astaxanthin and ketocarotenoids as femtograms (fg). Single carotenoids are normalized to 100 chlorophylls molecules. Data are expressed as means ± SD (*n* = 4). Statistical analysis was performed for each growth conditions: values marked with the same letters do not differ significantly (*P* < 0.05).

### Yield of astaxanthin in different growth conditions

**Figure 4:**
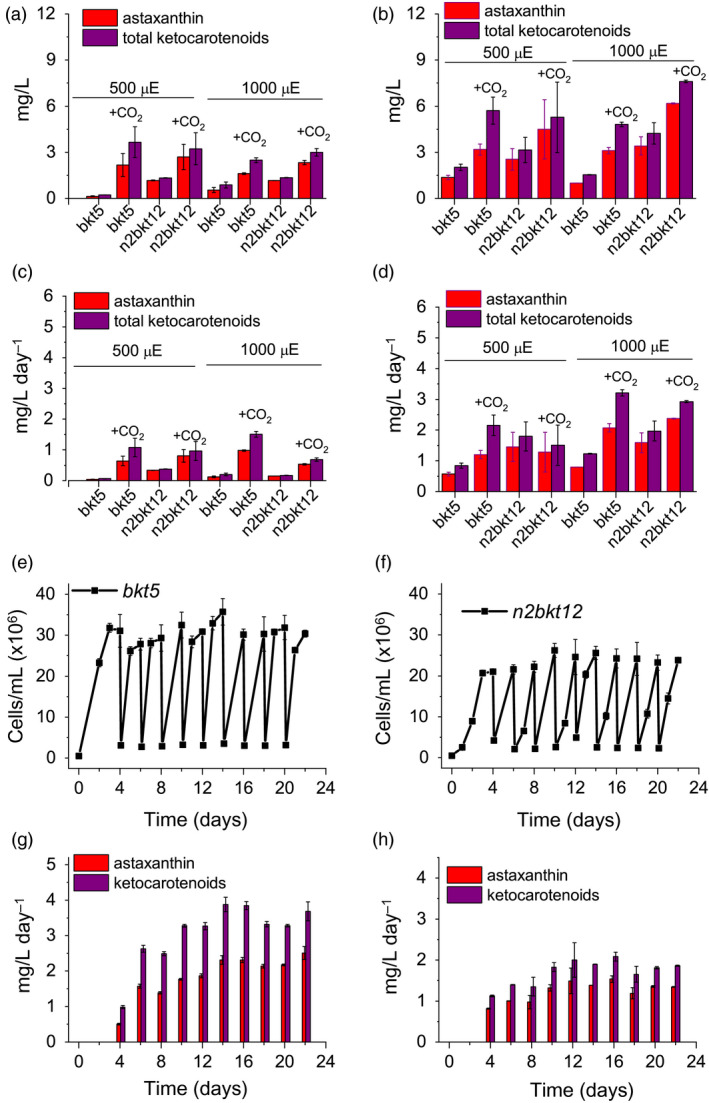
Astaxanthin and ketocarotenoid productivity in different growth conditions. Productivities of astaxanthin (red) and ketocarotenoids (purple) are presented as volumetric (mg/L) (a,b) and daily volumetric (mg/L/day) (c,d) productivities obtained from *bkt5* and *n2bkt12* mutants in autotrophic (a,c) or mixotrophic (b,d) growth. Cells were grown in HS or TAP media for autotrophic or mixotrophic growth, respectively, at 500 or 1000 µmol photons/m^2^/s with air bubbling or air plus 3% CO_2_ bubbling (CO_2_). Data were obtained by HPLC analysis. (e,f) Cell density of *bkt5* (e) or *n2bkt12* (f) strains cultivated in semi‐continuous mode in 500‐mL flasks at 500 µmol photons/m^2^/s in TAP with 3% CO_2_ bubbling and stirring. Cells were manually counted and diluted 10‐fold where they reached stationary phase. (g,h) Astaxanthin (red) and ketocarotenoid (purple) productivities obtained in semi‐continuous cultivation of *bkt5* (g) or *n2bkt12* (h) strains, as determined by HPLC analysis. Data are expressed as means ± standard deviation (*n* = 4).

Pigments accumulated in *bkt* and *n2bkt* lines were further analysed by high‐performance liquid chromatography (HPLC) to verify the accumulation of astaxanthin and other ketocarotenoids. HPLC chromatograms of UVM4 parental strain contain prominent peaks of chlorophylls a and b as well as of the carotenoids neoxanthin, violaxanthin, lutein and β‐carotene (Figure 3g). The transformed lines exhibit additional peaks (Figure 3g) due to the activity of the introduced *Cr*BKT enzyme. The additional peaks present in HPLC chromatograms in transformed lines were thus analysed by mass spectroscopy (Figure S3, Table S2) identifying three astaxanthin isoforms (peaks 8, 9 and 10), which according to elution time and absorption spectra (Figure S4) can be tentatively attributed to 3S,3′S trans‐astaxanthin, 3S,3′S 9‐cis‐astaxanthin and 3S,3′S 13‐cis‐astaxanthin (Holtin *et al.*, 2009; Yuan and Chen, 1997). Traces of other ketocarotenoids as adonirubin and canthaxanthin could also be identified by mass spectroscopy being eluted in peaks 11 and 12, respectively (Figure S3): canthaxanthin is produced by *Cr*BKT using β‐carotene as substrate (Figure 1), while adonirubin is an intermediate being formed during hydroxylation of canthaxanthin to astaxanthin or by *Cr*BKT using 3′OH‐echinenone as substrate (Ye *et al.*, 2007). Other possible ketocarotenoids produced by *Cr*BKT catalytic activity such as echinenone, 3′OH‐echinenone or adonixanthin could not be detected in *bkt5* or *n2bkt12* lines. In both parental strains, ketocarotenoids were never detected, while in *bkt5* and *n2bkt12* lines, they represent ~70% of the total cellular carotenoids, with astaxanthin as the major compound (Table 1). For all conditions tested with strain *bkt5*, reduced levels of other cellular carotenoids were observed. Violaxanthin underwent the most pronounced reduction of ~80%, and β‐carotene and neoxanthin were reduced by 65% and lutein 45%. Zeaxanthin was not decreased in *bkt5* although the amount of this carotenoid was low even in the parental strain (Table 1). *npq2* lines expressing *Cr*BKT exhibited strong reductions in zeaxanthin and β‐carotene contents, being both substrates from *Cr*BKT enzyme. In both, *bkt* and *n2bkt* lines, a clear decrease of the chlorophyll amount was evident with the strongest effect in autotrophy and high light conditions (in HS medium at 500 µmol/m^2^/s, Table 1) This result suggests that xanthophyll and β‐carotene reduction in favour of astaxanthin accumulation destabilize the chlorophyll content of photosynthetic complexes, similar to findings previously reported for *H. lacustris* (Mascia *et al.*, 2017). The highest ketocarotenoid content per cell was detected in *n2bkt* lines grown in mixotrophy at 100 or 500 µmol/m^2^/s, while mixotrophic growth at 500 µmol/m^2^/s promoted the highest values in *bkt* lines (Table 1). To further measure productivity of mutants expressing *Cr*BKT in different conditions, one line with the highest level of astaxanthin for each background, *bkt5* and *n2bkt12*, was selected for astaxanthin productivity analysis in small‐scale (80 mL) airlift photobioreactors. These two strains were cultivated at different light regimes (100 and 500 µmol/m^2^/s), in autotrophy (HS medium) or mixotrophy (TAP medium) and bubbling with air or 3% CO_2_. These experiments were performed in a semi‐continuous manner, with harvesting and culture dilution when stationary phase was reached (Figure 4). The experiment was continued until three cycles of dilution were repeated and the productivity of astaxanthin and ketocarotenoids was quantified as mg of pigments per litre per day of culture. In general, for both strains, mixotrophy was found to result in higher productivities compared to autotrophy (Figure 4a–d). Supplying CO_2_ to the culture clearly increased the productivity in all the different conditions especially when acetate was present (Figure 4a–d). For example, *bkt5* grown in TAP exhibited approximately double productivity rates of astaxanthin and ketocarotenoids when CO_2_ was added to TAP cultures at 500 and 1000 µmol/m^2^/s (Figure 4d). Light was observed to be important for the accumulation of astaxanthin in both *bkt5* and *n2bkt12* strains; overall, ketocarotenoid production increases were observed at 1000 µmol/m^2^/s compared to cells grown at 500 µmol/m^2^/s (Figure 4). The highest volumetric productivities obtained in these experiments were obtained for *bkt5* grown in TAP at 1000 µmol/m^2^/s with CO_2_ which produced ~2.07 ± 0.13 mg astaxanthin/L/day and 3.21 ± 0.10 mg total ketocarotenoids/L/day. The productivities in mg/L/day observed for *n2bkt12* were similar compared to the *bkt5*in these conditions (Figure 4d).

An additional experiment was performed in 600‐mL stirred flasks with TAP at 500 µmol/m^2^/s, in order to study the scalability of the productivity obtained with this mutant. The experiment was conducted for 3 weeks in continuous light (Figure 4e,f), and again, the productivity of astaxanthin and total ketocarotenoids was determined from the system during the cultivations (Figure 4g,h). Pigment productivities were quantified at every dilution, which was performed when the cells reached stationary phase. Both strains exhibited an adaption phase in the first days of growth in this system, followed by a phase where repetitive dilutions could be maintained for 3 weeks (Figure 4e,f). For strain *bkt5*, productivity values reached 3.88 ± 0.20 mg ketocarotenoids/L/day and 2.5 ± 0.18 mg astaxanthin/L/day (Figure 4g). A slower growth rate of *n2bkt12* led to a lower overall yield of ketocarotenoids and astaxanthin (Figure 4h). Although *n2bkt12* exhibited increased astaxanthin and ketocarotenoid productivity per cell, its slower growth rate hindered its competitiveness with the more rapid‐growing *bkt* line. Therefore, further productivity analyses focused only on *bkt5.*

**Figure 5:**
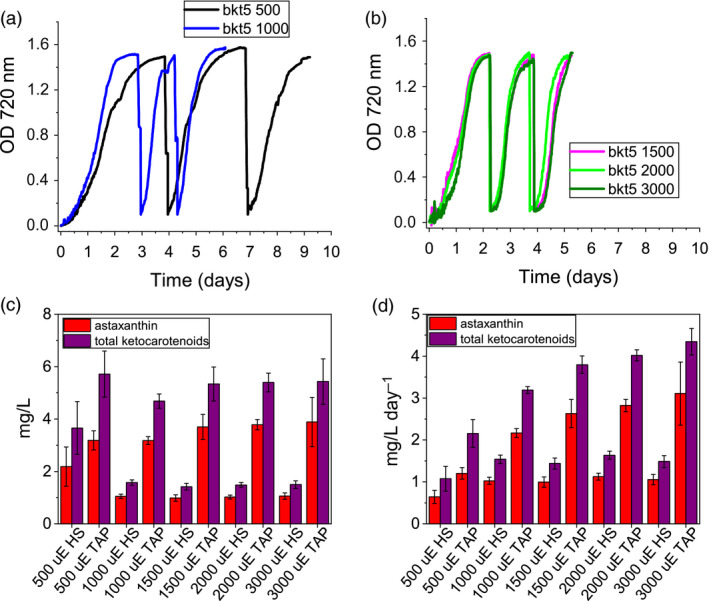
Astaxanthin and ketocarotenoids production by *bkt* lines in very high light conditions. (a,b) Growth curves of bkt lines cultivated with 3% CO_2_ bubbling at 500, 1000, 1500, 2000 or 3000 µmol photons/m^2^/s in mixotrophy conditions (TAP medium). Cells were manually diluted 10‐fold when the stationary phase was reached. Volumetric (c) and volumetric per day (d) productivities of astaxanthin (red) and ketocarotenoids (purple) obtained from *bkt5* mutant grown as reported in panels a and b. Data are expressed as means ± standard deviation (*n* = 4).

The productivity of astaxanthin and ketocarotenoid of the *bkt* strain was further investigated in 80‐mL airlift photobioreactors using conditions of autotrophy or mixotrophy, in the presence of 3% CO_2_, and with increasing irradiance up to 3000 µmol/m/s (Figure 5). In autotrophy, astaxanthin accumulation was reduced when irradiance was increased past 500 µmol/m/s, yet total ketocarotenoid amounts increased, suggesting different carotenogenesis dynamics in very high light conditions (Figure 5). In mixotrophy, both astaxanthin and total ketocarotenoid accumulation were higher compared to autotrophy conditions and partitioning between these carotenoid types was largely conserved across all irradiances (Figure 5c). Astaxanthin and ketocarotenoid productivity was similar at the different irradiances under autotrophic conditions: ~1 mg astaxanthin/L/day and ~1.5 mg ketocarotenoids/L/day. Mixotrophy was found to yield 2.6−3.1 mg astaxanthin/L/day and 3.7–4.3 mg ketocarotenoid/L/day at irradiances between 1500 and 3000 µmol/m/s.

### Extractability and bio‐accessibility of ketocarotenoids from cells

**Figure 6:**
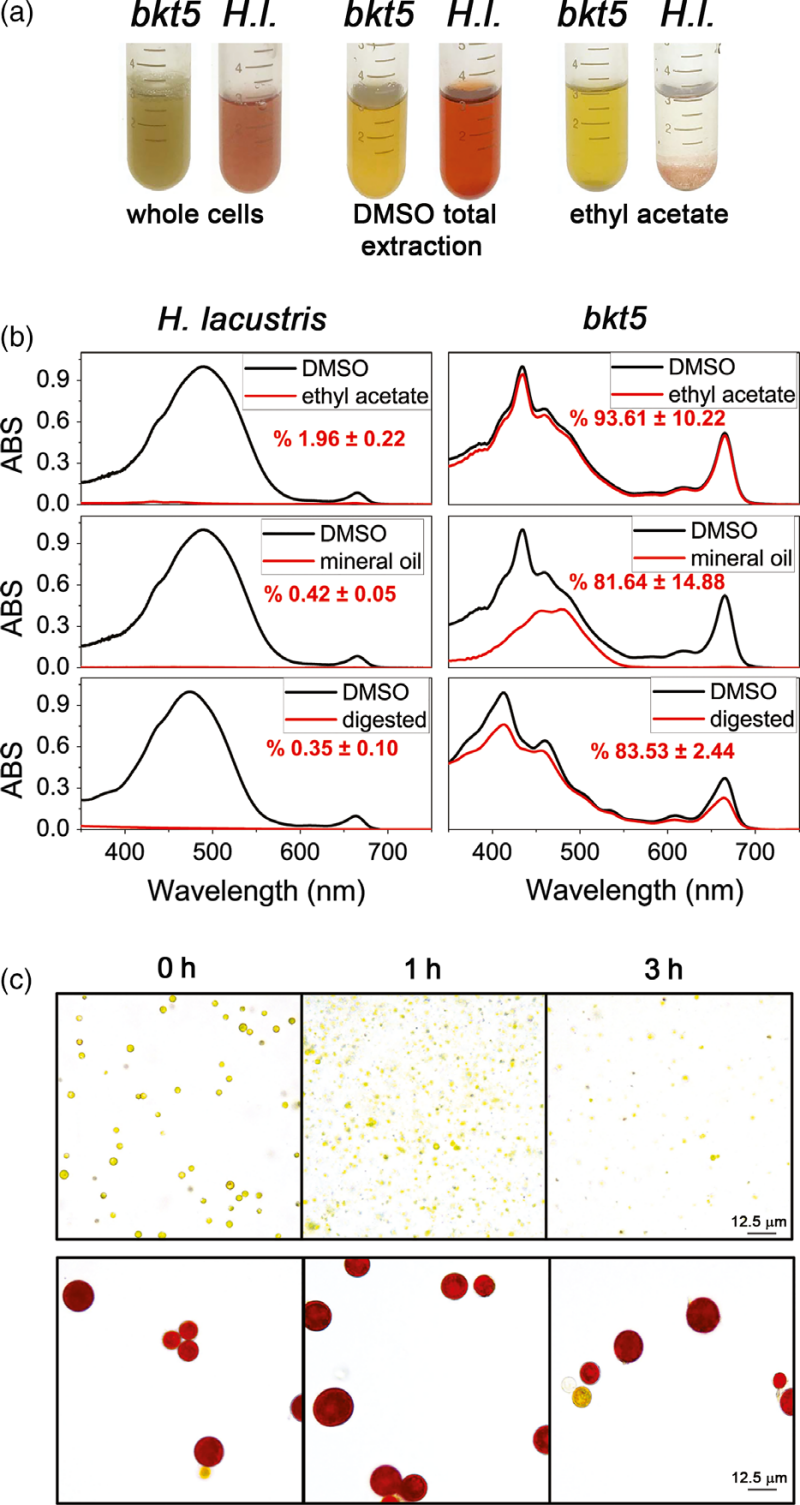
Extractability and bio‐accessibility of astaxanthin from *Haematococcus lacustris* and *Chlamydomonas reinhardtii* cells. (a) Aplanospore cysts of *H. lacustris* in ‘red phase’ and *C. reinhardtii bkt5* in liquid medium (left) were compared using different pigment extraction methods. DMSO is used as a control from total extraction of pigments from both hosts (centre) and ethyl acetate (right) or mineral oil were used. (b) Absorption spectra of pigments extracted from *H. lacustris* in red phase and *bkt5* with different solvents. Black traces are the absorption spectra of pigments obtained upon extraction in DMSO (positive control: maximum extraction); red traces show the pigments extracted with different methods. Spectra are normalized to the maximum absorption of the DMSO control. Inserts indicate the percentage of carotenoids extracted calculated from fitting analysis of the absorption spectra as described in Croce *et al. *(2000). Data are expressed as mean ± SD (*n* = 3). (c) Images of cells of *bkt5* and *H. lacustris* before (0 h) and after simulated gastro‐intestinal digestion (1 or 3 h).

One of the limitations in natural astaxanthin production from *H. lacustris* microalgae is the difficulty of pigment extraction from the cells which is hindered by the tough cell wall of the aplanospore cysts. These walls also limit the bio‐accessibility of *H. lacustris* astaxanthin as they are largely resistant to digestion (Sommer *et al.*, 1991). In order to evaluate a possible benefit in using *C. reinhardtii* for astaxanthin production over *H. lacustris*, the extractability of carotenoids from *C. reinhardtii bkt5* cells and *H. lacustris* cysts was compared by treating cells with solvent (ethyl acetate) or with mineral oil, a generally recognized as safe (GRAS) agent (Figure 6). Extraction with dimethyl sulphoxide (DMSO) was also used as a control as it has been previously reported to be the most effective method for total pigment extraction from *H. lacustris* (Zhekisheva *et al.*, 2002). Treating *H. lacustris* cells with either ethyl acetate or mineral oil gave an extremely low efficiency of astaxanthin extraction; less than 2% of total pigments could be extracted with these agents (Figure 6a,b). With *C. reinhardtii bkt5*, however, ethyl acetate extracted more than 93% of pigments from *C. reinhardtii* cells and the treatment with mineral oil extracted more than 80% of the carotenoids (Figure 6a,b). These experiments confirmed that extractability of pigments from the transformed *C. reinhardtii* cells is more readily achieved than from *H. lacustris*. Finally, the bio‐accessibility of astaxanthin produced in *C. reinhardtii* was compared to *H. lacustris* by simulating gastro‐intestinal digestion *in vitro* (Minekus *et al.*, 2014; Figure 6b,c). Cells were treated with simulated digestion fluids containing buffered solutions and enzymes, that is pepsin for the gastric phase and pancreatin for the intestinal phase. After 3 h, almost no pigment was extracted from *H. pluvialis* cysts by this method, while more than 80% of the pigments were extracted from *C. reinhardtii bkt5* (Figure 6b). These results indicate that cell wall‐deficient *C. reinhardtii* is a promising host organism for astaxanthin production as it is readily digestible and, consequently, the produced astaxanthin is more bio‐accessible.

## Discussion

The green microalga *C. reinhardtii* does not naturally accumulate ketocarotenoids; however, a beta‐carotene ketolase (*Cr*BKT) is found in its genome. *Cr*BKT has been shown to catalyse the conversion of carotenes into ketocarotenoids when heterologous expression of this gene was performed in other organisms; however, this sequence shows almost no detectible expression in the algal host itself (Figure 1b). Compared to BKT from other organisms, the *Cr*BKT contains a long peptide extension on its C‐terminus (Figure 1c), which appears to be an unfolded peptide region. Here, we took the *Cr*BKT sequence and used our recently developed transgene optimization strategy (Baier *et al.*, 2018b) to enable its re‐integration into the algal nuclear genome and overexpression. When *Cr*BKT was expressed, it caused the green algal cells to noticeably change colour from green to red, indicating the conversion of carotenoids to ketocarotenoids, with astaxanthin representing a major component of these. Our overexpression strategy of *Cr*BKT enzyme yielded better results compared to previous attempt where the BKT enzyme from *H. lacustris* expressed in *C. reinhardtii*: in the latter case indeed, only traces of ketocarotenoids as keto‐lutein or keto‐zeaxanthin were found (Leon *et al.*, 2007). We found the best combination of fusion partners and targeting peptides to be a fusion of the native gene, including its chloroplast targeting peptide, with the PsaD chloroplast targeting peptide and replacement of the C‐terminal amino acid extension with a GSG‐linker and YFP reporter (Figure 2c). The 116 amino acid long C‐terminal extension of the *Cr*BKT reduced overall astaxanthin productivity when included in expression constructs, however, did not completely abolish astaxanthin formation (Figure 2c). This suggests that the presence of this peptide alone is not responsible for its lack of expression in the algal host and that its low transcription rates likely play a larger role. Interestingly, the fusion of truncated BKT with YFP at the C‐terminus increased the production of ketocarotenoids (Figure 2c): likely, the presence of YFP at the C‐terminus somehow facilitates *Cr*BKT protein folding and/or further stabilizes it, although further work is required to verify this hypothesis. As the presence of astaxanthin did not perturb growth rates of either *UVM4‐bkt* or *npq2‐bkt* expression strains, at least in the conditions tested here (Figure 3), it is unclear what advantage repression of this native gene has given to the algal host. It is possible that a condition could exist where it is expressed: indeed, *Chlamydomonas nivalis*, a relative of *C. reinhardtii*, is known to produce astaxanthin as a photoprotective pigment under variable high light conditions on snow (Rezanka *et al.*, 2008). However, we did not find a condition where this gene was expressed to notable levels (based on FPKM) in the transcriptome databases investigated here (Figure 1b). Due to the very low rates of transcription, and the clear lack of ketocarotenoids in *C. reinhardtii*, we have called *Cr*BKT a pseudogene in this work.

Astaxanthin biosynthesis requires both a ketolase (BKT) and a hydroxylase (CHYB; Figure 1a). A *CHYB* gene is present in the nuclear genome of *C. reinhardtii* (Cre04.g215050) and it is expressed, even if to a lower extent compared to other genes related to carotenoid biosynthesis (Figure 1) (Lohr *et al.*, 2005). Re‐insertion of only *Cr*BKT into the nuclear genome of *C. reinhardtii* allowed the synthesis of high amounts of astaxanthin and other ketocarotenoids that normally do not accumulate in this organism (Figure 2). In contrast to previous work (Zheng *et al.*, 2014), the overexpression of *Cr*BKT alone was sufficient to induce ketocarotenoid formation in *C. reinhardtii*: these findings indicate that the native *Cr*CHYB is highly functional and able to participate in ketocarotenoid biosynthesis. When *Cr*BKT was overexpressed in the alga, astaxanthin became the major carotenoid (up to 50% of total carotenoids) with ketocarotenoids corresponding to ~70% of total carotenoids (Table 1). As the major substrate of *Cr*BKT is zeaxanthin, *Cr*BKT overexpression was also attempted in the *npq2* mutant, which is deficient in the zeaxanthin epoxidase and accumulates this as a terminal carotenoid (Niyogi *et al.*, 1997b). Indeed, astaxanthin accumulation per cell was higher in *npq2* than UVM4‐derived *bkt* lines (Table 1). However, *npq2* exhibits a slower growth rate than UVM4 (Figure 3), and although the growth was not affected by the presence of astaxanthin, overall volumetric productivities were higher with faster‐growing UVM4‐derived *bkt* lines (Figure 4).

The *bkt* mutants show a strong reduction of chlorophyll content of ~80% with respect to the parental line although remain photosynthetically active (Table 1, Figures 3b and 4). The xanthophylls lutein, violaxanthin and neoxanthin are ligands for the photosystem antenna subunits; β‐carotene is a ligand for both photosystem core complexes and antenna, while astaxanthin, in *H. lacustris*, is manly accumulated as a free form in the membrane, although it has been reported to bind photosystems (Mascia *et al.*, 2017). In a photosynthetic cell, the absence of carotenoids hinders photosystem assembly and reduces chlorophyll content (Cazzaniga *et al.*, 2016). It is possible that by rapidly converting carotenoids into ketocarotenoids, photosystem assembly is hindered, and lower chlorophyll amounts accumulate in the algal cell. Another possible reason for the observed decrease of chlorophyll contents in cells expressing *Cr*BKT could be that photosynthetic complexes, assembled without proper carotenoids, generate more ROS that damage photosynthetic membrane (Cazzaniga *et al.*, 2016); however, this hypothesis is less probable as no perturbation in growth rates were observed. In addition, high light intensities were tolerated by cells, and ketocarotenoids exhibit high antioxidant capacities which would likely protect membranes from ROS damage. In engineered plants, strains with an almost complete conversion of carotenoid to ketocarotenoids have been generated and showed slower growth rates, reduced photosynthetic parameters and increased photoinhibition (Fujii *et al.*, 2016; Hasunuma *et al.*, 2008; Zhong *et al.*, 2011). In *C. reinhardtii bkt* expression strains, ~30% of standard carotenoid and ~10%–20% of the standard chlorophyll were left in engineered strains and seemingly were enough to enable light energy absorption needed photosynthetic growth. The reddish‐brown phenotype (Figure 3a) of *bkt* transformants could be interesting for industrial application, as they are characterized by a decreased chlorophyll content which may allow better light penetration into dense algal cultures. Indeed, reduced antennae and chlorophyll containing phenotypes have previously been reported to promote increased algal culture productivity (Cazzaniga *et al.*, 2014; Melis, 2009).

*Haematococcus pluvialis* is currently considered the best natural source and the main commercial producing organism for astaxanthin as this alga can accumulate up to 4% of its dry weight as astaxanthin (Boussiba and Vonshak, 1991). Although *C. reinhardtii* strains presented in this work accumulate ketocarotenoids up to only 0.2% of their biomass, by investigating even minimal optimization of growth conditions, productivities of ketocarotenoids and astaxanthin could be reached up to 4.3 and 3.1 mg/L/day, respectively, when cells were grown in mixotrophy at irradiance 3000 µmol/m^2^/s bubbling 3% of CO_2_. *Chlamydomonas reinhardtii bkt5*reached high productivity even in autotrophic conditions, bubbling 3% of CO_2_, with ketocarotenoids and astaxanthin productivity of ~1.5 and ~1.0 mg/L/day, respectively, at irradiances between 1000 and 3000 µmol/m^2^/s. Due to the need of a two‐phase growth and astaxanthin induction, as well as a strong recalcitrant cell wall of astaxanthin containing aplanospores, the yield of astaxanthin from *H. lacustris* has been reported from 0.12 to 15 mg/L/day (Lopez *et al.*, 2006; Park *et al.*, 2014). Therefore, ketocarotenoid production for *C. reinhardtii bkt* mutants is within the same range of that of *H. lacustris*, however, requiring significantly fewer process parameters or extraction techniques, enabling also semi‐continuous or continuous production processes. Indeed, stable productivity of up to 3.88 mg ketocarotenoids/L/day and 2.5 mg astaxanthin/L/day was reached when *C. reinhardtii bkt5* cells were cultivated in semi‐continuous mode (Figure 4). Further optimization of *C. reinhardtii* cultivation, for example in higher‐density reactor concepts, will likely improve this efficiency.

Different techniques have been developed to disrupt *H. lacustris* cell walls and recover astaxanthin; these involve mechanical processes like high pressure or bead milling as well as the use of solvents, supercritical carbon dioxide or enzymatic digestion (Shah *et al.*, 2016). All these procedures increase the cost of astaxanthin bio‐production processes and exhibit a variable range of extraction efficiencies. *Chlamydomonas reinhardtii* strains commonly used for transformation and expression experiments generally lack cell walls. These strains still produce cell wall proteins; however, they are not able to assemble them on the cell surface and the proteinaceous wall proteins are instead secreted into the culture medium (Baier *et al.*, 2018a). Here, we used the UVM4 strain which is a derivative of the *cw15* line of cell wall‐deficient mutants (Neupert *et al.*, 2009). Due to the reduced cell wall, this strain is readily disrupted, enhancing the relative ease of pigment extraction from *bkt* expression lines. Astaxanthin extractability of *H. lacustris* aplanospores using ethyl acetate or mineral oil was below 1%, while pigments in *C. reinhardtii bkt* lines could be completely extracted under similar conditions (Figure 6). This increased extractability also means that pigments within engineered *C. reinhardtii* are more bio‐accessible, as the cyst state of astaxanthin containing *H. lacustris* may not be readily digested in target organisms (fish, livestock or humans). *In vitro* simulated digestion showed enhanced pigment extraction from *C. reinhardtii bkt5* compared to *H. lacustris*, indicating the engineered alga could be used directly in aquaculture and nutraceutical feed, without the need for prior pigment extraction (Figure 6b). It has been previously reported that astaxanthin produced in maize could be delivered to trout fillet when extracted in ethanol and vegetal oil, even if zeaxanthin and keto‐zeaxanthin were preferentially absorbed in the fish fillet (Breitenbach *et al.*, 2016). Direct use of algal biomass as a source of ketocarotenoid pigments for livestock and aquaculture could also benefit the quality of pigments delivered to these organisms as storage of carotenoids can lead to their degradation or oxidation. Further experimental work is required to investigate the possibility of using *C. reinhardtii*cells overexpressing BKT as a feed supplement.

Given it only requires the overexpression of a single ketolase to generate constitutive astaxanthin and ketocarotenoid generation in *C. reinhardtii*, it will likely be possible to transfer this biosynthesis rapidly into other more industrially cultivated algal strains. For example, certain *Chlorella* species have demonstrated robust outdoor growth, or the genetically amenable *Nannochloropsis* sp. may be alternatives where the production of astaxanthin can be scaled in existing infrastructures (Liu *et al.*, 2014; Lubián *et al.*, 2000). However, both of these algae also exhibit robust cell walls, which may limit overall process productivity. Indeed, *C. reinhardtii* has been shown amenable to scale up in airlift bioreactors even under outdoor conditions (Lauersen, 2018) and it may be that cultivation of cell wall‐deficient astaxanthin producing *C. reinhardtii* presents the best case of rapid growth and ease of pigment extractability. Moreover, additional biotechnological modifications can be attempted in *C. reinhardtii* in order to further boost carotenogenesis and astaxanthin productivity (Morikawa *et al.*, 2018). Given the robust activity of the *Cr*BKT, it may be likely that further customizations of the carotenoid biosynthesis can be readily achieved in this alga. Future engineering targets may seek to use existing carotenoids, or these new populations of ketocarotenoids, as even further targets for bioengineering optimization in this versatile algal host.

## Experimental procedures

### Algal cultivation and strain maintenance

*Chlamydomonas reinhardtii strain* UVM4 *was*graciously provided by Prof. Dr. Ralph Bock, and *npq2* (CC‐4101) was obtained by Chlamydomonas Resource Center (https://www.chlamycollection.org). Both strains were maintained on TAP agar plates or in liquid shake flasks at 25 °C with 100–150 µmol photons/m^2^/s of continuous white light. Growth tests were conducted using different systems: shaking flasks, stirring flasks or Multi‐Cultivator MC‐1000 (Photon Systems Instruments, Drasov, Czech Republic). Temperature was controlled to 25 °C, while light intensities were varied as indicated in the text. Tris‐acetate‐phosphate (TAP) or high‐salt (HS) minimal media were used for mixotrophic or photoautotrophic conditions, respectively, as described in the text (Harris and Harris, 2008). *Haematococcus lacustris* strain K‐0084 was obtained from Scandinavian Culture Collection of Algae & Protozoa and cultivated on BG‐11 medium as previously described (Scibilia *et al.*, 2015b).

### Design and cloning of expression cassettes

The *Cr*BKT (AY860820.1) found within the genome of *C. reinhardtii* was synthetically redesigned with codon optimization and intron spreading as recently described (Baier *et al.*, 2018b). The synthetic optimized *Cr*BKT gene coding sequence (CDS) was deprived of the last 345 bp and cloned into pOpt2_PsaD_mVenus_Paro vector (Wichmann *et al.*, 2018) to generate a protein which contains the *C. reinhardtii* photosystem I reaction centre subunit II (PsaD) chloroplast targeting peptide and a C‐terminal mVenus (YFP) fusion. Variations on this construct were generated by successive cloning within the pOpt2_PsaD_CrBKT_YFP_Paro vector as described in the text. For subcellular localization determination mediated of the BKT N‐terminal chloroplast targeting peptide, 102 and 120 bp from the 5′ region of the BKT coding for its N‐terminus was amplified and cloned into *Nde*I‐*Bgl*II sites of the pOpt2_PsaD_BKT_YFP_Paro. Additional details about design and cloning of expression cassettes can be found in Methods S1.

### *Chlamydomonas reinhardtii* transformation and mutant screening

Nuclear transformation was carried out by glass bead agitation as previously described (Kindle, 1990) using 10 μg of linearized plasmid DNA. Selection of transformants was done on TAP agar plates supplied by paromomycin (10 mg/L) for 5–7 days. Expressing colonies were pre‐screened visually for red coloration before further quantification.

### Fluorescence microscopy localization

Yellow fluorescent protein fluorescence imaging was performed as previously described (Lauersen *et al.*, 2016).

### Growth analysis

Parameters used for monitor growth were cell density and cell dry mass. Cell densities were measured using Countess II FL Automated Cell Counter (Thermo Fisher Scientific, Waltham, Massachusetts). Dry biomass was evaluated by overnight lyophilization of washed cell pellets and gravimetric determination.

### Pigment analyses

Pigments were extracted from intact cells using 80% acetone buffered with Na_2_CO_3_ and analysed by absorption spectra followed by curve fitting or by reverse‐phase HPLC. Absorption spectra were measured with Jasco V‐550 UV/VIS spectrophotometer as described in Cinque *et al. *(2000). Spectra were fitted as described in Croce *et al. *(2002) introducing in the fitting method the astaxanthin absorption form in acetone 80%: considering the similar absorption of astaxanthin and canthaxanthin, the results obtained by fitting of pigment extracts absorption spectra were considered as representative to total ketocarotenoids. Reverse‐phase HPLC was conducted as described in Lagarde *et al. *(2000) and Scibilia *et al. *(2015a). HPLC system equipped with a C18 column using a 15‐min gradient of ethyl acetate (0%–100%) in acetonitrile–water–triethylamine (9 : 1 : 0.01, vol/vol/vol) at a flow rate of 1.5 mL/min was used. Pigment detection was conducted with a Thermo Fisher 350–750 nm diode array detector. Ketocarotenoid peaks were identified by comparing retention times and spectra to commercially available standards (CaroteNature GmbH, Münsingen, Switzerland). Different astaxanthin isomers were identified according to literature (Holtin *et al.*, 2009; Yuan and Chen, 1997).

For LC mass spectroscopy measurements, single peaks were collected from HPLC separation. Sample were dried and resuspended in acetonitrile and loaded on HPLC 1260 (Agilent Technologies, Waldbronn, Germany) in tandem with a QTOF mass spectrometer was used for the analysis. The QTOF‐MS was implemented with an electrospray ion source with Agilent Jet Stream technology operating in positive ionization mode. Data acquisition was performed in full scan mode in the mass range of 200–1000 *m*/*z*.

### Extractability and simulated digestion of ketocarotenoids in *Haematococcus lacustris* and *Chlamydomonas reinhardtii*

Same weight of dried cells of *H. lacustris* and *bkt5* were resuspended in water and treated with ethyl acetate or mineral oil for 20 min at room temperature and subsequently subject to centrifugation. Extracted pigments present in the supernatant were recovered, and spectra were recorded to determine relative extractability. Treatment was repeated once for ethyl acetate and three times for mineral oil. The simulate digestion was performed following the protocol described by Minekus *et al. *(2014) with some modification. 0.1 g of freeze‐dried cells (LIO‐5P, five pascal) were resuspended in 1 mL of simulated gastric fluid and stirred for 1 h at 37 °C, and then, 2 mL of simulated intestinal fluid was added to the samples. After further 2 h at as above, samples were centrifuged for 3 min at 3000 ***g*** to pellet intact cells and isolate the supernatant digested fraction. Pigments were extracted with acetone from the digested fraction and spectra recorded.

## Conflict of interest

The authors declare no conflict of interest.

## Author contributions

M.B. conceived the work. M.B., L.W. and K.J.L supervised experiments. F.P. performed or contributed to all the experiments herein reported. S.C. contributed to experiments reported in Table 1, Figures 3, 4, 5, 6 and Figures S2–S4. K.J.L, L.W. and T.B. designed strategies for BKT overexpression in *C. reinhardtii* and contributed to generation and selection of overexpressing *C. reinhardtii* strains. G.Z. and F.Z. contributed to experiments reported in Figure 6. S.C. and M.B. wrote the manuscript with contributions from all the authors. All the authors discussed the results, contributed to data interpretation and commented on the manuscript.

## Supporting information


**Figure S1** Western blot and immunodetection of BKT proteins fused with YFP.
**Figure S2** Microscopy images of *Haematococcus lacustris* and *Chlamydomonas reinhardtii* BKT overexpressing strain (*bkt 5*).
**Figure S3** Mass spectroscopy of ketocarotenoids accumulated in *bkt* strains.
**Figure S4** Absorption spectra of major ketocarotenoids accumulated in *bkt *strains.
**Table S1** Percentage of colonies with visible phenotype upon transformation with *bkt* expression vectors.
**Table S2** Chromatographic, spectroscopic and mass properties of identified carotenoids.

**Methods S1** Gene engineering and cloning.

## References

[pbi13364-bib-0001] Alvarez, V. , Rodríguez‐Sáiz, M. , de la Fuente, J.L. , Gudiña, E.J. , Godio, R.P. , Martín, J.F. and Barredo, J.L. (2006) The crtS gene of Xanthophyllomyces dendrorhous encodes a novel cytochrome‐P450 hydroxylase involved in the conversion of beta‐carotene into astaxanthin and other xanthophylls. Fungal Genet. Biol. 43, 261–272. 16455271 10.1016/j.fgb.2005.12.004

[pbi13364-bib-0002] Baier, T. , Kros, D. , Feiner, R.C. , Lauersen, K.J. , Muller, K.M. and Kruse, O. .(2018a) Engineered fusion proteins for efficient protein secretion and purification of a human growth factor from the green microalga *Chlamydomonas reinhardtii* . ACS Synth. Biol. 7, 2547–2557. 30296377 10.1021/acssynbio.8b00226

[pbi13364-bib-0003] Baier, T. , Wichmann, J. , Kruse, O. and Lauersen, K.J. (2018b) Intron‐containing algal transgenes mediate efficient recombinant gene expression in the green microalga *Chlamydomonas reinhardtii* . Nucleic Acids Res. 46, 6909–6919. 30053227 10.1093/nar/gky532 PMC6061784

[pbi13364-bib-0004] Bennedsen, M. , Wang, X. , Willen, R. , Wadstrom, T. and Andersen, L.P. (1999) Treatment of *H. pylori* infected mice with antioxidant astaxanthin reduces gastric inflammation, bacterial load and modulates cytokine release by splenocytes. Immunol. Lett. 70, 185–189. 10656672 10.1016/s0165-2478(99)00145-5

[pbi13364-bib-0005] Boussiba, S. and Vonshak, A. (1991) Astaxanthin accumulation in the green alga *Haematococcus pluvialis*1. Plant Cell Physiol. 32, 1077–1082.

[pbi13364-bib-0006] Breitenbach, J. , Nogueira, M. , Farré, G. , Zhu, C. , Capell, T. , Christou, P. , Fleck, G. *et al*. (2016) Engineered maize as a source of astaxanthin: processing and application as fish feed. Transgenic Res. 25, 785–793. 27520497 10.1007/s11248-016-9971-3

[pbi13364-bib-0007] Britton, G. (1995) Structure and properties of carotenoids in relation to function. FASEB J. 9, 1551–1558. 8529834

[pbi13364-bib-0008] Bubrick, P. (1991) Production of astaxanthin from Haematococcus. Bioresour. Technol. 38, 237–239.

[pbi13364-bib-0009] Cazzaniga, S. , Dall'Osto, L. , Szaub, J. , Scibilia, L. , Ballottari, M. , Purton, S. and Bassi, R. (2014) Domestication of the green alga *Chlorella sorokiniana*: reduction of antenna size improves light‐use efficiency in a photobioreactor. Biotechnol. Biofuels, 7, 157. 10.1186/s13068-014-0157-z. 25352913 PMC4210543

[pbi13364-bib-0010] Cazzaniga, S. , Bressan, M. , Carbonera, D. , Agostini, A. and Dall'Osto, L. (2016) Differential roles of carotenes and xanthophylls in photosystem I photoprotection. Biochemistry, 55, 3636–3649. 27290879 10.1021/acs.biochem.6b00425

[pbi13364-bib-0011] Chen, J.‐H. , Liu, L. and Wei, D. (2017) Enhanced production of astaxanthin by Chromochloris zofingiensis in a microplate‐based culture system under high light irradiation. Bioresour. Technol. 245, 518–529. 28898852 10.1016/j.biortech.2017.08.102

[pbi13364-bib-0012] Cinque, G. , Croce, R. and Bassi, R. (2000) Absorption spectra of chlorophyll a and b in Lhcb protein environment. Photosynth. Res. 64, 233–242. 16228461 10.1023/A:1006467617697

[pbi13364-bib-0013] Couso, I. , Cordero, B.F. , Vargas, M.A. and Rodriguez, H. (2012) Efficient heterologous transformation of *Chlamydomonas reinhardtii* npq2 mutant with the zeaxanthin epoxidase gene isolated and characterized from Chlorella zofingiensis. Mar. Drugs, 10, 1955–1976. 23118714 10.3390/md10091955 PMC3475266

[pbi13364-bib-0014] Croce, R. , Cinque, G. , Holzwarth, A.R. and Bassi, R. (2000) The Soret absorption properties of carotenoids and chlorophylls in antenna complexes of higher plants. Photosynth. Res. 64, 221–231. 16228460 10.1023/A:1006455230379

[pbi13364-bib-0015] Croce, R. , Canino, G. , Ros, F. and Bassi, R. (2002) Chromophore organization in the higher‐plant photosystem II antenna protein CP26. Biochemistry, 41, 7334–7343. 12044165 10.1021/bi0257437

[pbi13364-bib-0016] Cunningham, F.X. and Gantt, E. (1998) Genes and enzymes of carotenoid biosynthesis in plants. Ann. Rev. Plant Physiol. Plant Mol. Biol. 49, 557–583. 15012246 10.1146/annurev.arplant.49.1.557

[pbi13364-bib-0017] Cunningham, F.X. Jr and Gantt, E. (2011) Elucidation of the pathway to astaxanthin in the flowers of *Adonis aestivalis* . Plant Cell, 23, 3055–3069. 21862704 10.1105/tpc.111.086827 PMC3180810

[pbi13364-bib-0018] Edge, R. , McGarvey, D.J. and Truscott, T.G. (1997) The carotenoids as anti‐oxidants – a review. J. Photochem. Photobiol. B Biol. 41, 189–200. 10.1016/s1011-1344(97)00092-4 9447718

[pbi13364-bib-0019] Fujii, R. , Yamano, N. , Hashimoto, H. , Misawa, N. and Ifuku, K. (2016) Photoprotection vs. photoinhibition of photosystem II in transplastomic lettuce (*Lactuca sativa*) dominantly accumulating astaxanthin. Plant Cell Physiol. 57, 1518–1529. 26644463 10.1093/pcp/pcv187

[pbi13364-bib-0020] Gerster, H. (1997) The potential role of lycopene for human health. J. Am. College Nutr. 16, 109–126. 10.1080/07315724.1997.10718661 9100211

[pbi13364-bib-0021] Gross, G.J. and Lockwood, S.F. (2004) Cardioprotection and myocardial salvage by a disodium disuccinate astaxanthin derivative (Cardax). Life Sci. 75, 215–224. 15120573 10.1016/j.lfs.2003.12.006

[pbi13364-bib-0022] Grossman, A.R. , Lohr, M. and Im, C.S. (2004) *Chlamydomonas reinhardtii* in the landscape of pigments. Annu. Rev. Genet. 38, 119–173. 15568974 10.1146/annurev.genet.38.072902.092328

[pbi13364-bib-0023] Harada, H. , Maoka, T. , Osawa, A. , Hattan, J. , Kanamoto, H. , Shindo, K. , Otomatsu, T. *et al*. (2014) Construction of transplastomic lettuce (*Lactuca sativa*) dominantly producing astaxanthin fatty acid esters and detailed chemical analysis of generated carotenoids. Transgenic Res. 23, 303–315. 24287848 10.1007/s11248-013-9750-3

[pbi13364-bib-0024] Harker, M. and Hirschberg, J. (1997) Biosynthesis of ketocarotenoids in transgenic cyanobacteria expressing the algal gene for beta‐C‐4‐oxygenase, crtO. FEBS Lett. 404, 129–134. 9119049 10.1016/s0014-5793(97)00110-5

[pbi13364-bib-0025] Harris, E.H. (2008) Introduction to Chlamydomonas and its Laboratory Use. San Diego, CA: Academic Press.

[pbi13364-bib-0026] Hasunuma, T. , Miyazawa, S. , Yoshimura, S. , Shinzaki, Y. , Tomizawa, K. , Shindo, K. , Choi, S.K. *et al*. (2008) Biosynthesis of astaxanthin in tobacco leaves by transplastomic engineering. Plant J. 55, 857–868. 18494855 10.1111/j.1365-313X.2008.03559.x

[pbi13364-bib-0027] Henke, N. , Heider, S. , Peters‐Wendisch, P. and Wendisch, V. (2016) Production of the marine carotenoid astaxanthin by metabolically engineered *Corynebacterium glutamicum* . Mar. Drugs, 14, 124. 27376307 10.3390/md14070124 PMC4962014

[pbi13364-bib-0028] Holtin, K. , Kuehnle, M. , Rehbein, J. , Schuler, P. , Nicholson, G. and Albert, K. (2009) Determination of astaxanthin and astaxanthin esters in the microalgae *Haematococcus pluvialis* by LC‐(APCI)MS and characterization of predominant carotenoid isomers by NMR spectroscopy. Anal. Bioanal. Chem. 395, 1613–1622. 19466394 10.1007/s00216-009-2837-2

[pbi13364-bib-0029] Huang, J.‐C. , Zhong, Y.‐J. , Liu, J. , Sandmann, G. and Chen, F. (2013) Metabolic engineering of tomato for high‐yield production of astaxanthin. Metab. Eng. 17, 59–67. 23511430 10.1016/j.ymben.2013.02.005

[pbi13364-bib-0030] Hussein, G. , Sankawa, U. , Goto, H. , Matsumoto, K. and Watanabe, H. (2006) Astaxanthin, a carotenoid with potential in human health and nutrition. J. Nat. Prod. 69, 443–449. 16562856 10.1021/np050354+

[pbi13364-bib-0031] Jayaraj, J. , Devlin, R. and Punja, Z. (2008) Metabolic engineering of novel ketocarotenoid production in carrot plants. Transgenic Res. 17, 489–501. 17682834 10.1007/s11248-007-9120-0

[pbi13364-bib-0032] Jyonouchi, H. , Sun, S. and Gross, M. (1995) Effect of carotenoids on in vitro immunoglobulin production by human peripheral blood mononuclear cells: astaxanthin, a carotenoid without vitamin A activity, enhances in vitro immunoglobulin production in response to a T‐dependent stimulant and antigen. Nutri. Cancer, 23, 171–183. 10.1080/01635589509514373 7644386

[pbi13364-bib-0033] Kang, C.D. and Sim, S.J. (2008) Direct extraction of astaxanthin from *Haematococcus* culture using vegetable oils. Biotechnol. Lett. 30, 441–444. 17972016 10.1007/s10529-007-9578-0

[pbi13364-bib-0034] Kildegaard, K.R. , Adiego‐Perez, B. , Domenech Belda, D. , Khangura, J.K. , Holkenbrink, C. and Borodina, I. (2017) Engineering of Yarrowia lipolytica for production of astaxanthin. Synth. Syst. Biotechnol. 2, 287–294. 29552653 10.1016/j.synbio.2017.10.002 PMC5851922

[pbi13364-bib-0035] Kim, J.H. , Park, J.J. , Lee, B.J. , Joo, M.K. , Chun, H.J. , Lee, S.W. and Bak, Y.T. (2016) Astaxanthin inhibits proliferation of human gastric cancer cell lines by interrupting cell cycle progression. Gut Liver, 10, 369–374. 26470770 10.5009/gnl15208 PMC4849689

[pbi13364-bib-0036] Kindle, K.L. (1990) High‐frequency nuclear transformation of Chlamydomonas reinhardtii. Proc. Natl. Acad. Sci. USA, 87, 1228–1232. 2105499 10.1073/pnas.87.3.1228 PMC53444

[pbi13364-bib-0037] Kobayashi, M. , Kurimura, Y. , Kakizono, T. , Nishio, N. and Tsuji, Y. (1997) Morphological changes in the life cycle of the green alga *Haematococcus pluvialis* . J. Ferment. Bioeng. 84, 94–97.

[pbi13364-bib-0038] Krieger‐Liszkay, A. (2005) Singlet oxygen production in photosynthesis. J. Exp. Bot. 56, 337–346. 10.1093/jxb/erh237 15310815

[pbi13364-bib-0039] Kull, O. and Pfander, H. (1995) List of new carotenoids. In Carotenoids: Isolation and Analysis ( Britton, G. , Liaaen‐Jensen , S. and Pfander , H. , eds), pp. 295–317. Basel: Birkauser Publishing.

[pbi13364-bib-0040] Lagarde, D. , Beuf, L. and Vermaas, W. (2000) Increased production of zeaxanthin and other pigments by application of genetic engineering techniques to Synechocystis sp. strain PCC 6803. Appl. Environ. Microbiol. 66, 64–72. 10618204 10.1128/aem.66.1.64-72.2000 PMC91786

[pbi13364-bib-0041] Lauersen, K.J. (2018) Eukaryotic microalgae as hosts for light‐driven heterologous isoprenoid production. Planta, 249, 155–180. 30467629 10.1007/s00425-018-3048-x

[pbi13364-bib-0042] Lauersen, K.J. , Kruse, O. and Mussgnug, J.H. (2015) Targeted expression of nuclear transgenes in *Chlamydomonas reinhardtii* with a versatile, modular vector toolkit. Appl. Microbiol. Biotechnol. 99, 3491–3503. 25586579 10.1007/s00253-014-6354-7

[pbi13364-bib-0043] Lauersen, K.J. , Baier, T. , Wichmann, J. , Wordenweber, R. , Mussgnug, J.H. , Hubner, W. , Huser, T. *et al*. (2016) Efficient phototrophic production of a high‐value sesquiterpenoid from the eukaryotic microalga *Chlamydomonas reinhardtii* . Metab. Eng. 38, 331–343. 27474353 10.1016/j.ymben.2016.07.013

[pbi13364-bib-0044] Lauersen, K.J. , Wichmann, J. , Baier, T. , Kampranis, S.C. , Pateraki, I. , Moller, B.L. and Kruse, O. (2018) Phototrophic production of heterologous diterpenoids and a hydroxy‐functionalized derivative from *Chlamydomonas reinhardtii* . Metab. Eng. 49, 116–127. 30017797 10.1016/j.ymben.2018.07.005

[pbi13364-bib-0045] Leon, R. , Couso, I. and Fernandez, E. (2007) Metabolic engineering of ketocarotenoids biosynthesis in the unicelullar microalga *Chlamydomonas reinhardtii* . J. Biotechnol. 130, 143–152. 17433482 10.1016/j.jbiotec.2007.03.005

[pbi13364-bib-0046] Li, J. , Zhu, D. , Niu, J. , Shen, S. and Wang, G. (2011) An economic assessment of astaxanthin production by large scale cultivation of *Haematococcus pluvialis* . Biotechnol. Adv. 29, 568–574. 21497650 10.1016/j.biotechadv.2011.04.001

[pbi13364-bib-0047] Liu, J. , Sun, Z. , Gerken, H. , Liu, Z. , Jiang, Y. and Chen, F. (2014) Chlorella zofingiensis as an alternative microalgal producer of astaxanthin: biology and industrial potential. Mar. Drugs, 12, 3487–3515. 24918452 10.3390/md12063487 PMC4071588

[pbi13364-bib-0048] Lohr, M. , Im, C.S. and Grossman, A.R. (2005) Genome‐based examination of chlorophyll and carotenoid biosynthesis in *Chlamydomonas reinhardtii* . Plant Physiol. 138, 490–515. 15849308 10.1104/pp.104.056069 PMC1104202

[pbi13364-bib-0049] Lopez, M.C. , Sanchez Edel, R. , Lopez, J.L. , Fernandez, F.G. , Sevilla, J.M. , Rivas, J. , Guerrero, M.G. *et al*. (2006) Comparative analysis of the outdoor culture of *Haematococcus pluvialis* in tubular and bubble column photobioreactors. J. Biotechnol. 123, 329–342. 16406158 10.1016/j.jbiotec.2005.11.010

[pbi13364-bib-0050] Lotan, T. and Hirschberg, J. (1995) Cloning and expression in *Escherichia coli* of the gene encoding beta‐C‐4‐oxygenase, that converts beta‐carotene to the ketocarotenoid canthaxanthin in *Haematococcus pluvialis* . FEBS Lett. 364, 125–128. 7750556 10.1016/0014-5793(95)00368-j

[pbi13364-bib-0051] Lubián, L.M. , Montero, O. , Moreno‐Garrido, I. , Huertas, I.E. , Sobrino, C. , González‐del Valle, M. and Parés, G. (2000) Nannochloropsis (Eustigmatophyceae) as source of commercially valuable pigments. J. Appl. Phycol. 12, 249–255.

[pbi13364-bib-0052] Mann, V. , Harker, M. , Pecker, I. and Hirschberg, J. (2000) Metabolic engineering of astaxanthin production in tobacco flowers. Nat. Biotechnol. 18, 888–892. 10932161 10.1038/78515

[pbi13364-bib-0053] Mascia, F. , Girolomoni, L. , Alcocer, M.J.P. , Bargigia, I. , Perozeni, F. , Cazzaniga, S. , Cerullo, G. *et al*. (2017) Functional analysis of photosynthetic pigment binding complexes in the green alga *Haematococcus pluvialis* reveals distribution of astaxanthin in photosystems. Sci. Rep. 7, 16319. 29176710 10.1038/s41598-017-16641-6 PMC5701160

[pbi13364-bib-0054] Melis, A. (2009) Solar energy conversion efficiencies in photosynthesis: minimizing the chlorophyll antennae to maximize efficiency. Plant Sci. 177, 272–280.

[pbi13364-bib-0055] Merchant, S.S. , Prochnik, S.E. , Vallon, O. , Harris, E.H. , Karpowicz, S.J. , Witman, G.B. , Terry, A. *et al*. (2007) The Chlamydomonas genome reveals the evolution of key animal and plant functions. Science, 318, 245–250. 17932292 10.1126/science.1143609 PMC2875087

[pbi13364-bib-0056] Miki, W. (1991) Biological functions and activities of animal carotenoids. Pure Appl. Chem. 63, 141–146.

[pbi13364-bib-0057] Mimuro, M. and Katoh, T. (1991) Carotenoids in photosynthesis: Absorption, transfer and dissipation of light energy. Pure Appl. Chem. 63, 123–130.

[pbi13364-bib-0058] Minekus, M. , Alminger, M. , Alvito, P. , Ballance, S. , Bohn, T. , Bourlieu, C. , Carriere, F. *et al*. (2014) A standardised static in vitro digestion method suitable for food ‐ an international consensus. Food Funct. 5, 1113–1124. 24803111 10.1039/c3fo60702j

[pbi13364-bib-0059] Misawa, N. , Satomi, Y. , Kondo, K. , Yokoyama, A. , Kajiwara, S. , Saito, T. , Ohtani, T. *et al*. (1995) Structure and functional analysis of a marine bacterial carotenoid biosynthesis gene cluster and astaxanthin biosynthetic pathway proposed at the gene level. J. Bacteriol. 177, 6575–6584. 7592436 10.1128/jb.177.22.6575-6584.1995 PMC177511

[pbi13364-bib-0060] Miura, Y. , Kondo, K. , Saito, T. , Shimada, H. , Fraser, P.D. and Misawa, N. (1998) Production of the carotenoids lycopene, beta‐carotene, and astaxanthin in the food yeast *Candida utilis* . Appl. Environ. Microbiol. 64, 1226–1229. 9546156 10.1128/aem.64.4.1226-1229.1998 PMC106133

[pbi13364-bib-0061] Møller, A.P. , Biard, C. , Blount, J.D. , Houston, D.C. , Ninni, P. , Saino, N. and Surai, P.F. (2000) Carotenoid‐dependent signals: indicators of foraging efficiency, immunocompetence or detoxification ability? Avian Poultry Biol. Rev. 11, 137–159.

[pbi13364-bib-0062] Morikawa, T. , Uraguchi, Y. , Sanda, S. , Nakagawa, S. and Sawayama, S. (2018) Overexpression of DnaJ‐like chaperone enhances carotenoid synthesis in *Chlamydomonas reinhardtii* . Appl. Biochem. Biotechnol. 184, 80–91. 28612271 10.1007/s12010-017-2521-5

[pbi13364-bib-0063] Naguib, Y.M. (2000) Antioxidant activities of astaxanthin and related carotenoids. J. Agric. Food Chem. 48, 1150–1154. 10775364 10.1021/jf991106k

[pbi13364-bib-0064] Nakada, T. and Ota, S. (2016) What is the correct name for the type of *Haematococcus* Flot. (Volvocales, Chlorophyceae)? Taxon, 65, 343–348.

[pbi13364-bib-0065] Neupert, J. , Karcher, D. and Bock, R. (2009) Generation of *Chlamydomonas* strains that efficiently express nuclear transgenes. Plant J. 57, 1140–1150. 19036032 10.1111/j.1365-313X.2008.03746.x

[pbi13364-bib-0066] Niyogi, K.K. , Bjorkman, O. and Grossman, A.R. (1997a) Chlamydomonas xanthophyll cycle mutants identified by video imaging of chlorophyll fluorescence quenching. Plant Cell, 9, 1369–1380. 12237386 10.1105/tpc.9.8.1369 PMC157004

[pbi13364-bib-0067] Niyogi, K.K. , Björkman, O. and Grossman, A.R. (1997b) Chlamydomonas xanthophyll cycle mutants identified by video imaging of chlorophyll fluorescence quenching. Plant Cell, 9, 1369–1380. 12237386 10.1105/tpc.9.8.1369 PMC157004

[pbi13364-bib-0068] Nogueira, M. , Enfissi, E.M.A. , Martínez Valenzuela, M.E. , Menard, G.N. , Driller, R.L. , Eastmond, P.J. , Schuch, W. *et al*. (2017) Engineering of tomato for the sustainable production of ketocarotenoids and its evaluation in aquaculture feed. Proc. Natl. Acad. Sci. USA, 114, 10876–10881. 28973873 10.1073/pnas.1708349114 PMC5642710

[pbi13364-bib-0069] Palozza, P. , Torelli, C. , Boninsegna, A. , Simone, R. , Catalano, A. , Mele, M.C. and Picci, N. (2009) Growth‐inhibitory effects of the astaxanthin‐rich alga *Haematococcus pluvialis* in human colon cancer cells. Cancer Lett. 283, 108–117. 19423215 10.1016/j.canlet.2009.03.031

[pbi13364-bib-0070] Park, J.C. , Choi, S.P. , Hong, M.E. and Sim, S.J. (2014) Enhanced astaxanthin production from microalga, *Haematococcus pluvialis* by two‐stage perfusion culture with stepwise light irradiation. Bioprocess Biosyst. Eng. 37, 2039–2047. 24700132 10.1007/s00449-014-1180-y

[pbi13364-bib-0071] Park, S.Y. , Binkley, R.M. , Kim, W.J. , Lee, M.H. and Lee, S.Y. (2018) Metabolic engineering of *Escherichia coli* for high‐level astaxanthin production with high productivity. Metab. Eng. 49, 105–115. 30096424 10.1016/j.ymben.2018.08.002

[pbi13364-bib-0072] Rasala, B.A. , Barrera, D.J. , Ng, J. , Plucinak, T.M. , Rosenberg, J.N. , Weeks, D.P. , Oyler, G.A. *et al*. (2013) Expanding the spectral palette of fluorescent proteins for the green microalga *Chlamydomonas reinhardtii* . Plant J. 74, 545–556. 23521393 10.1111/tpj.12165

[pbi13364-bib-0073] Rezanka, T. , Nedbalova, L. , Sigler, K. and Cepak, V. (2008) Identification of astaxanthin diglucoside diesters from snow alga *Chlamydomonas nivalis* by liquid chromatography‐atmospheric pressure chemical ionization mass spectrometry. Phytochemistry, 69, 479–490. 17681561 10.1016/j.phytochem.2007.06.025

[pbi13364-bib-0074] Romero‐Campero, F.J. , Perez‐Hurtado, I. , Lucas‐Reina, E. , Romero, J.M. and Valverde, F. (2016) ChlamyNET: a Chlamydomonas gene co‐expression network reveals global properties of the transcriptome and the early setup of key co‐expression patterns in the green lineage. BMC Genomics, 17, 227. 26968660 10.1186/s12864-016-2564-y PMC4788957

[pbi13364-bib-0075] Scibilia, L. , Girolomoni, L. , Berteotti, S. , Alboresi, A. and Ballottari, M. (2015a) Photosynthetic response to nitrogen starvation and high light in *Haematococcus pluvialis* . Algal Res. 12, 12–181.

[pbi13364-bib-0076] Shah, M.M.R. , Liang, Y. , Cheng, J.J. and Daroch, M. (2016) Astaxanthin‐Producing green microalga *Haematococcus pluvialis*: from single cell to high value commercial products. Front. Plant Sci. 7, 531. 10.3389/fpls.2016.00531. 27200009 PMC4848535

[pbi13364-bib-0077] Sommer, T.R. , Potts, W.T. and Morrissy, N.M. (1991) Utilization of microalgal astaxanthin by rainbow trout (*Oncorhynchus mykiss*). Aquaculture, 94, 79–88.

[pbi13364-bib-0078] Stalberg, K. , Lindgren, O. , Ek, B. and Hoglund, A.S. (2003) Synthesis of ketocarotenoids in the seed of *Arabidopsis thaliana* . Plant J. 36, 771–779. 14675443 10.1046/j.1365-313x.2003.01919.x

[pbi13364-bib-0079] Tan, C.‐P. , Zhao, F.‐Q. , Su, Z.‐L. , Liang, C.‐W. and Qin, S. (2007) Expression of β‐carotene hydroxylase gene (crtR‐B) from the green alga *Haematococcus pluvialis* in chloroplasts of *Chlamydomonas reinhardtii* . J. Appl. Phycol. 19, 347–355.

[pbi13364-bib-0080] Uchiyama, K. , Naito, Y. , Hasegawa, G. , Nakamura, N. , Takahashi, J. and Yoshikawa, T. (2002) Astaxanthin protects beta‐cells against glucose toxicity in diabetic db/db mice. Redox Rep. 7, 290–293. 12688512 10.1179/135100002125000811

[pbi13364-bib-0081] Wichmann, J. , Baier, T. , Wentnagel, E. , Lauersen, K.J. and Kruse, O. (2018) Tailored carbon partitioning for phototrophic production of (E)‐alpha‐bisabolene from the green microalga *Chlamydomonas reinhardtii* . Metab. Eng. 45, 211–222. 29258965 10.1016/j.ymben.2017.12.010

[pbi13364-bib-0082] Wu, H. , Niu, H. , Shao, A. , Wu, C. , Dixon, B.J. , Zhang, J. , Yang, S. *et al*. (2015) Astaxanthin as a potential neuroprotective agent for neurological diseases. Mar. Drugs, 13, 5750–5766. 26378548 10.3390/md13095750 PMC4584352

[pbi13364-bib-0083] Ye, R.W. , Yao, H. , Stead, K. , Wang, T. , Tao, L. , Cheng, Q. , Sharpe, P.L. *et al*. (2007) Construction of the astaxanthin biosynthetic pathway in a methanotrophic bacterium *Methylomonas* sp. strain 16a. J. Ind. Microbiol. Biotechnol. 34, 289–299. 17205350 10.1007/s10295-006-0197-x

[pbi13364-bib-0084] Yuan, J.‐P. and Chen, F. (1997) Identification of astaxanthin isomers in *Haematococcus lacustris* by HPLC‐photodiode array detection. Biotechnol. Tech. 11, 455–459.

[pbi13364-bib-0085] Yuan, J.P. , Peng, J. , Yin, K. and Wang, J.H. (2011) Potential health‐promoting effects of astaxanthin: a high‐value carotenoid mostly from microalgae. Mol. Nutr. Food Res. 55, 150–165. 21207519 10.1002/mnfr.201000414

[pbi13364-bib-0086] Yunus, I.S. , Wichmann, J. , Wordenweber, R. , Lauersen, K.J. , Kruse, O. and Jones, P.R. (2018) Synthetic metabolic pathways for photobiological conversion of CO2 into hydrocarbon fuel. Metab. Eng. 49, 201–211. 30144559 10.1016/j.ymben.2018.08.008

[pbi13364-bib-0087] Zhang, L. and Wang, H. (2015) Multiple mechanisms of anti‐cancer effects exerted by astaxanthin. Mar. Drugs, 13, 4310–4330. 26184238 10.3390/md13074310 PMC4515619

[pbi13364-bib-0088] Zhekisheva, M. , Boussiba, S. , Khozin‐Goldberg, I. , Zarka, A. and Cohen, Z. (2002) Accumulation of oleic acid in *Haematococcus pluvialis* (chlorophyceae) under nitrogen starvation or high light is correlated with that of astaxanthin esters1. J. Phycol. 38, 325–331.

[pbi13364-bib-0089] Zheng, K. , Wang, C. , Xiao, M. , Chen, J. , Li, J. and Hu, Z. (2014) Expression of bkt and bch genes from *Haematococcus pluvialis* in transgenic Chlamydomonas. Science China. Life Sci. 57, 1028–1033. 10.1007/s11427-014-4729-8 25209726

[pbi13364-bib-0090] Zhong, Y.J. , Huang, J.C. , Liu, J. , Li, Y. , Jiang, Y. , Xu, Z.F. , Sandmann, G. *et al*. (2011) Functional characterization of various algal carotenoid ketolases reveals that ketolating zeaxanthin efficiently is essential for high production of astaxanthin in transgenic Arabidopsis. J Exp. Bot. 62, 3659–3669. 21398427 10.1093/jxb/err070 PMC3130182

