# Supplementary material for: Turning a green alga red: engineering astaxanthin biosynthesis by intragenic pseudogene revival in Chlamydomonas reinhardtii
Source: Plant Biotechnol J. 2020 Mar 31;18(10):2053–67. doi: 10.1111/pbi.13364 (PMC7540493; doi:10.1111/pbi.13364)

Supplementary Information

Methods S1

The *Cr*BKT (AY860820.1) found within the genome of *C. reinhardtii* was synthetically redesigned with codon optimization and intron spreading as recently described (Baier et al., 2018b). The synthetic optimized *Cr*BKT gene coding sequence (CDS) was deprived of the last 345 bp and a small region was added to make a C-terminal GSG-linker prior to optimization and the synthetic gene produced by GeneArt (Germany). The synthetic *Cr*BKT sequence was cloned between *Bam*HI-*Bgl*II into the pOpt2_PsaD_mVenus_Paro vector (Wichmann et al., 2018) to generate a protein which contains the *C. reinhardtii* photosystem I reaction centre subunit II (PsaD) chloroplast targeting peptide and a C-terminal mVenus (YFP) fusion. The empty vector served as a control. The vector confers resistance to paromomycin from a second *aphVIII* expression cassette (Lauersen et al., 2015). Variations on this construct were generated by successive cloning within the pOpt2_PsaD_CrBKT_YFP_Paro vector. The *Cr*BKT was cloned into the same vector but without PsaD target peptide in order to obtain a *Cr*BKT protein targeted with endogenous transit peptide. To test the C-terminal amino acid extension, the whole protein was generated by PCR fusion of a 345 bp region to its C-terminus. First, the C-terminal region was chemically synthesized including last 30 bp of BKT previously generated in order to allow primer binding. Then two different fragments were amplified: one containing BKT sequence using pOpt2_PsaD_BKT_YFP as template (primer: for: 5'-GGCCGGATCCGGCCCCGGCATCCAGCCCACCAGCG-3', rev: 5'-CCGCGGTCGCGGCAGCCGCGGCGGGCACGGGCAGGGGGCCGGGGGCCAGGGCGGCGCCGCGGGCGATC-3') while the second contained the BKT C-terminal region using synthetic sequence as template (primer for: 5'-GATCGCCCGCGGCGCCGCCCTG-3', rev: 5'-GGCCAGATCTGCCGCTGCCGGCCATCACGCCCACGGGGGCCAGC-3'). The two fragments were then used as template for an additional amplification to fuse these elements using additional primers for: 5'-GGCCGGATCCGGCCCCGGCATCCAGCCCACCAGCG-3' and rev: 5'-GGCCAGATCTGCCGCTGCCGGCCATCACGCCCACGGGGGCCAGC-3'. The amplified sequence was cloned into pOpt2_PsaD_BKT_YFP_Paro in the *Bam*HI-*Bgl*II position.
pOpt2_PsaD_BKT without YFP was generated by removing YFP sequence using *Zra*I and *Eco*RV enzymes from pOpt2_PsaD_BKT_YFP. pOpt_PsaD_BKT2x_YFP was generated by first PCR amplification of BKT sequence to remove the 5' 150 nucleotides coding for the N-terminal targeting peptide using pOpt2_PsaD_BKT_YFP_Paro as template (primer: for 5'-GGCCGGATCCAAGCTGTGGCAGCGCCAGTACCACCTG-3', rev: 5'-GGCCAGATCTGCCGCTGCCGGCCATCACGCCCACGGGGGCCAGC-3'). This sequence was inserted into a pOpt2 vector generating pOpt2_PsaD_-50aaBKT_YFP which could then be combined with the pOpt2_PsaD_BKT_YFP_Paro vector by compatible *Bam*HI-*Bgl*II overhangs to generate the pOpt2_PsaD_2xBKT_YFP_Paro. For subcellular localization determination mediated of the BKT N-terminal chloroplast targeting peptide, 102 and 120 bp from the 5' region of the BKT coding for its N-terminus was amplified and cloned into *Nde*I-*Bgl*II sites of the pOpt2_PsaD_BKT_YFP_Paro (primer for: 5'-GGCCGGATCCGGCCCCGGCATCCAGCCCACCAGCG-3' rev:amino acid 34 for 102bp and rev:amino acid 40 for 120bp).
All cloning described here was performed using FastDigest restriction enzymes (Thermo Scientific) and the Rapid DNA Dephos & Ligation Kit (Roche) following manufacturer's instructions. PCR was conducted using Q5® High Fidelity DNAPolymerase (NEB). DNA fragments were separated in 2% (w/v) agarose gels and purified using the peqGOLD Gel Extraction Kit (VWR). Heat-shock transformation of chemically competent *Escherichia coli* DH5α cells was performed for all vectors followed by selection on LB-agar plates with ampicillin as a selection marker. Colonies were evaluated by colony PCR followed by plasmid isolation from overnight culture using peqGOLD

Plasmid Miniprep Kit I (VWR). All sequences were confirmed by Sanger sequencing (Sequencing Core Facility, CeBiTec, Bielefeld University).

Table S1. Percentage of colonies with visible phenotype upon transformation with *bkt* expression vectors. The percentage of colonies on plates with visible phenotype upon transformation with the different expression vectors carrying *bkt* gene was calculated from the ratio between brown/green colonies among the colonies surviving the antibiotic selection. Negative control with expression vectors carrying *yfp* gene only are reported. Errors are reported as standard deviations.

	vector	altered colour/total
**UVM4**	PsaD_YFP	0.00% ± 0%
	PsaD_BKT_YFP	16.40% ± 0.80%
	PsaD_BKT_Ct_YFP	14.50% ± 3.50%
	PsaD_BKT	13.30% ± 9.20%
	BKT_YFP	17.70% ± 6.50%
	PsaD_BKT2x_YFP	16.00% ± 12.00%
**npq2**	PsaD_YFP	0.00% ± 0%
	PsaD_BKT_YFP	17.95% ± 8%

Table S2. Chromatographic, spectroscopic and mass properties of identified carotenoids. First three columns indicate peak numbers, retention times (RT) and absorption peaks (λ max) of different carotenoids analysed by HPLC as shown in figure 3g. Single purified ketocarotenoids (indicated with *) were further analysed by LC Mass spectroscopy. Retention times for LC-MS, m/z value (corresponding to $[M+H]^+$ values), chemical formula generated and the difference between the theoretical and measured mass value (Δ ppm) are reported.

	HPLC peak	HPLC RT (min)	λ max (nm)	LC-MS RT (min)	m/z	chemical formula generated by LC-MS	Δ (ppm)
neoxanthin	1	4.83	415,438,466	-	-	-	-
violaxanthin	2	5.85	418,442,470	-	-	-	-
*3S 3S' trans astaxanthin	8	6.35	478	1.60	597.4	$C_{40} H_{52} O_4$	0.77
*3S 3S' 9 cis astaxanthin	9	7.01	470	1.60	597.4	$C_{40} H_{52} O_4$	0.52
*3S 3S' 13 cis astaxanthin	10	7.48	371,468	1.60	597.4	$C_{40} H_{52} O_4$	0.33
lutein	3	7.83	420,447,475	-	-	-	
*adonirubin (Phoenicoxanthin)	11	8.09	474	2.02	581,4	$C_{40} H_{52} O_3$	-1.24
*cantaxanthin	12	8.52	476	2.42	565.4	$C_{40} H_{53} O_2$	0.15
β carotene	6	13.35	428,455,478	-	-	-	-

Figure S1. Western blot and immunodetection of BKT proteins fused with YFP. Protein extracts from transformant lines, as reported on the top of figure, we loaded in SDS-PAGE gel. Proteins were separated by 12% Tris-glycine-SDS-PAGE. Separated proteins were stained using colloidal Coomassie. Brilliant Blue G-250 or analyzed by immunodetection on nitrocellulose membranes using a HRP-linked rabbit-anti-GFP antibody (Thermo Scientific, A10260) and Pierce™ ECL Western Blotting substrates (Thermo Scientific). Coomassie staining is reported on the bottom of the figure as loading control.

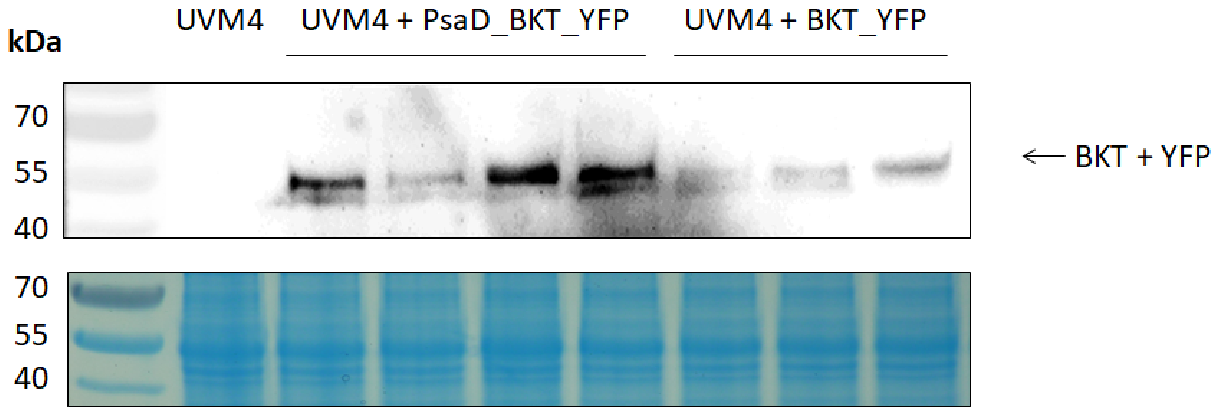

Figure S2. Microscopy images of *H. lacustris* and *C. reinhardtii* BKT overexpressing strain (*bkt5*). *bkt5* strain was generated by transforming *C. reinhardtii* UVM4 strain with PsaD_BKT_YFP construct (Figure 2). *H. lacustris* cells are presented in green and red phases. Red phase was obtained by high light treatment and nitrogen starvation for 5 days.

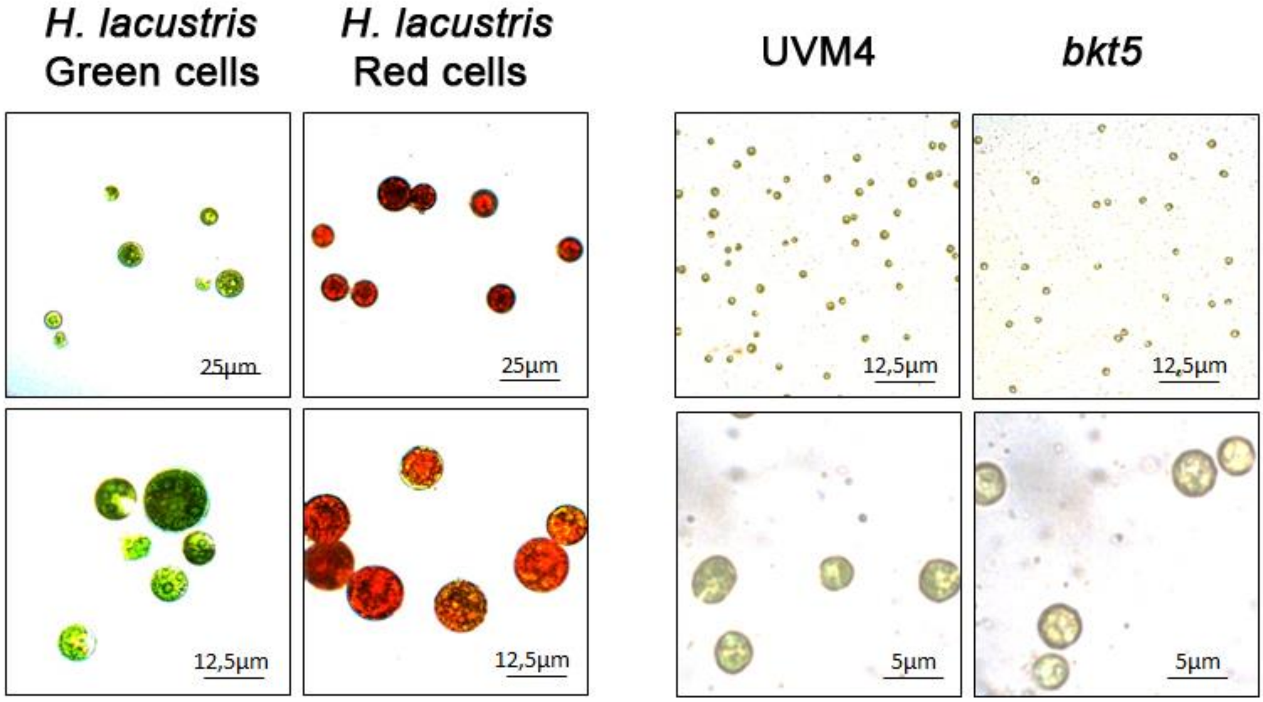

Figure S3. Mass spectroscopy of ketocarotenoids accumulated in *bkt* strains. Mass spectra of major ketocarotenoids present in *bkt* mutants. Peak numbers correspond to those present on HPLC chromatogram in Figure 3.

Astaxanthin (Peaks 8, 9, 10)

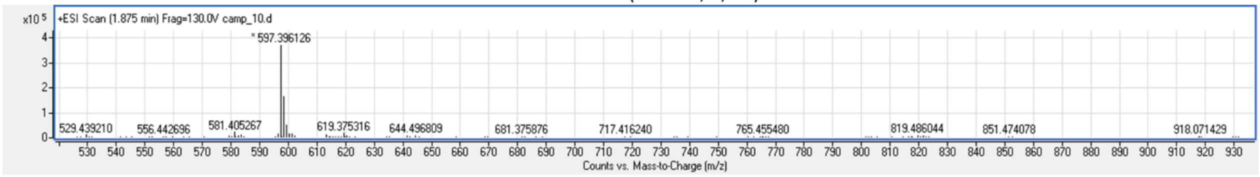

Adonirubin (Peak 11)

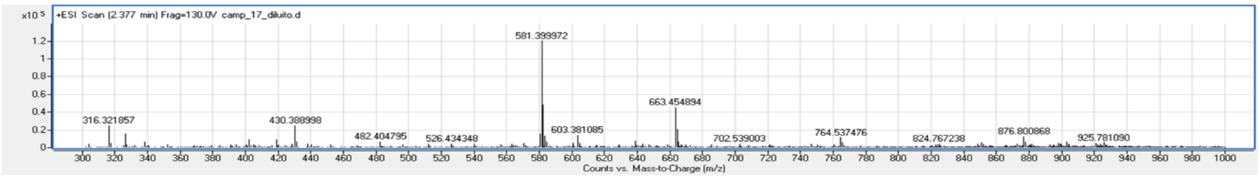

Canthaxanthin (Peak 12)

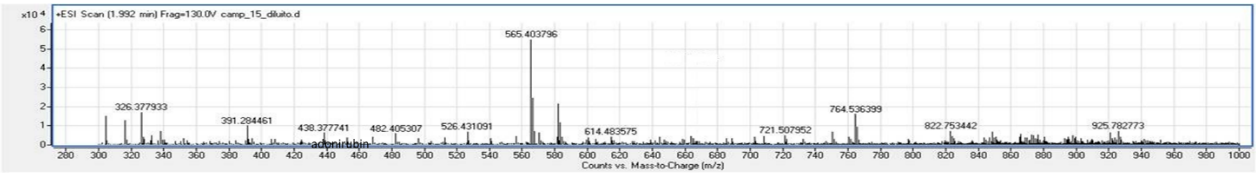

Figure S4. Absorption spectra of major ketocarotenoids accumulated in *bkt* strains. Absorbance (a-e) spectra of major ketocarotenoids present in bkt mutants resolved by HPLC analysis. Peak numbers correspond to those present on chromatogram in Figure 3

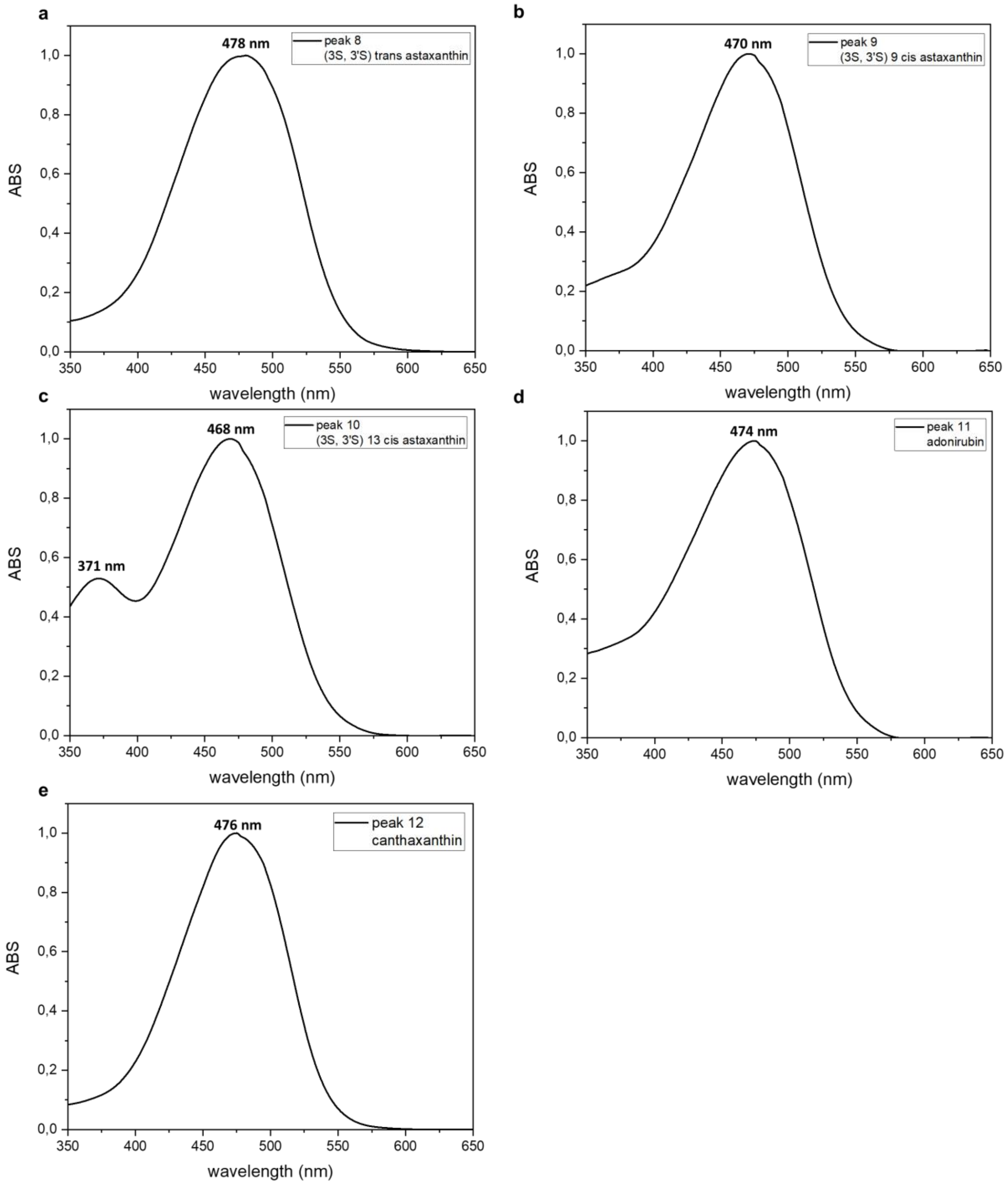

Supplement: Supplementary file 1 — Methods S1 Gene engineering and cloning. [file PBI-18-2053-s001.pdf]
